# Multiple mechanisms shape the relationship between pathway and duration of focal seizures

**DOI:** 10.1093/braincomms/fcac173

**Published:** 2022-07-06

**Authors:** Gabrielle M Schroeder, Fahmida A Chowdhury, Mark J Cook, Beate Diehl, John S Duncan, Philippa J Karoly, Peter N Taylor, Yujiang Wang

**Affiliations:** CNNP Lab, Interdisciplinary Computing and Complex BioSystems Group, School of Computing, Newcastle University, Newcastle upon Tyne, United Kingdom; UCL Queen Square Institute of Neurology, Queen Square, London, United Kingdom; Graeme Clark Institute and St Vincent’s Hospital, University of Melbourne, Melbourne, VIC, Australia; Seer Medical Pty Ltd, Melbourne, VIC, Australia; UCL Queen Square Institute of Neurology, Queen Square, London, United Kingdom; UCL Queen Square Institute of Neurology, Queen Square, London, United Kingdom; Graeme Clark Institute and St Vincent’s Hospital, University of Melbourne, Melbourne, VIC, Australia; Department of Biomedical Engineering, University of Melbourne, Melbourne, VIC, Australia; Seer Medical Pty Ltd, Melbourne, VIC, Australia; CNNP Lab, Interdisciplinary Computing and Complex BioSystems Group, School of Computing, Newcastle University, Newcastle upon Tyne, United Kingdom; Faculty of Medical Sciences, Newcastle University, Newcastle upon Tyne, United Kingdom; UCL Queen Square Institute of Neurology, Queen Square, London, United Kingdom; CNNP Lab, Interdisciplinary Computing and Complex BioSystems Group, School of Computing, Newcastle University, Newcastle upon Tyne, United Kingdom; Faculty of Medical Sciences, Newcastle University, Newcastle upon Tyne, United Kingdom; UCL Queen Square Institute of Neurology, Queen Square, London, United Kingdom

**Keywords:** seizure, duration, pathway, intracranial EEG, NeuroVista

## Abstract

A seizure’s electrographic dynamics are characterized by its spatiotemporal evolution, also termed dynamical ‘pathway’, and the time it takes to complete that pathway, which results in the seizure’s duration. Both seizure pathways and durations have been shown to vary within the same patient. However, it is unclear whether seizures following the same pathway will have the same duration or if these features can vary independently. We compared within-subject variability in these seizure features using (i) epilepsy monitoring unit intracranial EEG (iEEG) recordings of 31 patients (mean: 6.7 days, 16.5 seizures/subject), (ii) NeuroVista chronic iEEG recordings of 10 patients (mean: 521.2 days, 252.6 seizures/subject) and (iii) chronic iEEG recordings of three dogs with focal-onset seizures (mean: 324.4 days, 62.3 seizures/subject). While the strength of the relationship between seizure pathways and durations was highly subject-specific, in most subjects, changes in seizure pathways were only weakly to moderately associated with differences in seizure durations. The relationship between seizure pathways and durations was strengthened by seizures that were ‘truncated’ versions, both in pathway and duration, of other seizures. However, the relationship was weakened by seizures that had a common pathway, but different durations (‘elasticity’), or had similar durations, but followed different pathways (‘semblance’). Even in subjects with distinct populations of short and long seizures, seizure durations were not a reliable indicator of different seizure pathways. These findings suggest that seizure pathways and durations are modulated by multiple different mechanisms. Uncovering such mechanisms may reveal novel therapeutic targets for reducing seizure duration and severity.

## Teaser

Schroeder *et al*. investigated the relationship between epileptic seizure duration and the sequence of activity patterns during a seizure (termed ‘seizure pathway’). They found multiple mechanisms modulating the relationship, and concluded that the duration of a seizure is generally not a reliable indicator of its pathway.

## Graphical Abstract

**Graphical abstract:**
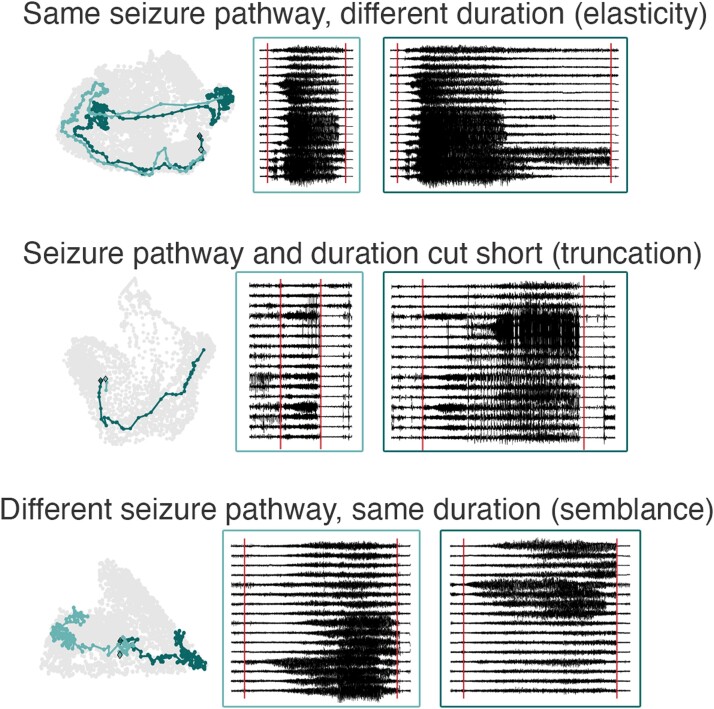

## Introduction

Many health conditions are challenging to treat due to changes in symptoms and disease severity over time.^1–3^ Recent research has emphasized that temporal fluctuations are also an important consideration in focal epilepsy, as seizures can change over time within the same patient.^4–9^ Specifically, a patient’s spatiotemporal seizure dynamics can vary in two main ways. First, the evolution of pathological activity, as measured by EEG recordings, can differ from seizure to seizure. These seizure evolutions can be described mathematically with various computational models, including functional networks^4,10^ and neural mass models,^7^ that capture specific dynamical seizure properties. Using such approaches, each seizure evolution can be conceptualized as a pathway through the chosen feature space.^4,7,11–13^ Second, each seizure may be characterized by its duration, the time that elapses from its electrographic start to finish.^14–17^ Together, these features describe both the sequence of brain activity during a seizure as well as the time that it takes to complete that sequence.

Both seizure pathways and seizure durations are related to seizure clinical symptoms, severity, and clinical management.^4,15–18^ For example, seizure duration is featured in several seizure severity scales.^18^ Certain types of diversity in seizure pathways, such as multifocal onsets^19–21^ and variable recruitment patterns,^22,23^ are associated with worse outcomes following epilepsy surgery. Additionally, seizure prediction is more challenging in patients who have distinct populations of short and long seizures.^6^ Despite the clinical relevance of seizure pathways and durations, little is known about how these features interact. In particular, it is unclear whether variability in seizure duration arises purely from changes in seizure pathways, or whether pathways and durations can vary independently within the same patient.

It is conceivable that two seizures with distinct pathways must also have distinct durations. In an analogy, where two passengers embark on a bus at the same stop, the passenger riding for more stops (longer pathway) will also necessarily have a longer journey time (longer duration). Some previous studies suggest that seizure pathways and durations are linked in a similar manner. First, seizure duration often differs between different International League Against Epilepsy clinical seizure types,^24^ which are also associated with changes in functional networks.^4,10,25^ More severe seizure types have also been reported to last longer; for example, focal seizures that progress to bilateral tonic–clonic seizures tend to have longer durations than seizures that remain focal,^15,16^ and focal seizures tend to be longer if they involve loss of awareness.^16,17^ Meanwhile, analysis of chronic iEEG recordings suggests that seizures with different durations have similar onsets, but different terminations.^7^ Additionally, there is evidence that distinct populations of short and long seizures correspond to different seizure pathways with characteristic durations in some patients.^6,7^ These findings suggest that seizure pathways and durations may be linked within patients, with different seizure durations serving as a proxy for different seizure pathways and *vice versa*.

It is also possible that seizure duration is modulated independently of the seizure’s pathway. In our analogy above, it is possible to travel the same bus route (same pathway) on two different days, with a longer journey time (longer duration) on the day with more traffic. Similarly, two seizures could potentially follow the same pathway, but have different durations due to different rates of progression (e.g. by ‘dwelling’ in particular EEG activity patterns). In a rodent model, Wenzel *et al.*^26^ found seizures with consistent recruitment patterns and different rates of seizure spread at a neuronal level, a characteristic termed ‘elasticity’. To our knowledge, no studies have quantitatively explored such temporal flexibility in seizure pathways in human patients. Nonetheless, within-patient seizures with consistent firing patterns, but small changes in duration, have been observed,^27^ suggesting that elasticity in the same seizure pathway may also occur in humans. This mechanism could potentially lead to variable durations among seizures with the same pathway.

The relationship between seizure pathways and durations has been difficult to investigate due to the lack of an objective measure for comparing seizure pathways. We addressed this need by proposing an approach for quantitatively comparing within-patient seizure pathways, which we used to investigate variability in seizure functional network evolutions.^4^ In the present study, we used the same approach to explore whether variability in seizure pathways is linked to variability in seizure durations. We hypothesized that certain generalizable mechanisms strengthen or weaken the association between seizure pathways and duration, creating varying levels of association within each patient. Our quantitative comparison of seizure pathways allowed us to recognize similar pathways even if they progressed at different rates. Thus, we could determine whether two seizures shared the same pathway, even if their durations differed. We also extended our analysis to include long-term recordings from NeuroVista patients^28^ and dogs,^29,30^ allowing us to analyze the relationship between pathways and durations in subjects with a higher number of recorded seizures that occurred over longer timescales.

## Methods

### Subjects and seizure data

This work analyzed seizures from the following three cohorts of subjects:

1. *Epilepsy monitoring unit* (*EMU*) *patients:* 31 patients with refractory focal epilepsy whose continuous iEEG recordings were acquired during presurgical evaluation at the Mayo Clinic (MC) (12 patients), the Hospital of the University of Pennsylvania (HUP) (1 patient), or the University College London Hospital (UCLH) (18 patients). MC and HUP patient data are available on the IEEG Portal, www.ieeg.org,^31,32^ and all IEEG Portal patients gave consent for their anonymized iEEG to be available through this database. The iEEG recordings of the UCLH patients were anonymized, exported, and analyzed under the approval of the Newcastle University Ethics Committee (reference number 6887/2018). The same EMU cohort and seizure data were used in our previous analysis of seizure variability.^4^ Additional information about each patient and the analyzed seizures is shown in Supplementary Table 1.
2. *NeuroVista patients*: Seizures from 10 NeuroVista patients were included to analyze seizure variability over longer timescales in patients with focal epilepsy. The NeuroVista seizure iEEG data,^7^ which includes 12 patients, were used for this analysis. Patients of NeuroVista 2 and NeuroVista 4 were removed from our analysis due to low numbers of analyzable seizures. The patients and collection of their chronic iEEG data are described in detail in Cook *et al.*^28^ Briefly, all patients had refractory focal epilepsy and experienced 2–12 seizures per month. For the NeuroVista seizure prediction study, each patient was implanted with 16 surface iEEG electrodes over the brain quadrant thought to contain the epileptogenic zone. Additional patient details are provided in Supplementary Table 2.
3. *Dogs:* To explore seizure variability over longer timescales and in non-human subjects, iEEG was also analyzed from three dogs with focal-onset seizures due to naturally occurring canine epilepsy. The dogs underwent prolonged recordings to test a novel implantable electrographic recording device^29,30^ (recording data available on the IEEG Portal, www.ieeg.org^31,32^). Metadata for the dogs are provided in Supplementary Table 3.

For all cohorts, seizures were required to have clear electrographic correlates and durations of at least 10 s. We excluded seizures with noisy or large missing segments. Subclinical seizures were included in the analysis. For NeuroVista patients, seizures with clinical manifestations and corresponding iEEG changes (referred to as ‘Type 1’ seizures in the previous literature^6,7^) and seizures with iEEG changes comparable to Type 1 seizures, but without confirmed clinical manifestations (previously referred to as ‘Type 2’ seizures) were included in the analysis.

For EMU patients, seizure onsets and terminations were marked by the corresponding clinical teams. For NeuroVista patients, seizure onsets and terminations were marked by clinical staff, with seizure detection and classification aided by using patient diaries, audio recordings, and a seizure detection algorithm.^6^ For dogs, seizure onsets were marked by expert readers and an algorithm was used to detect seizure termination (see Supplementary Text 3).

### Intracranial EEG preprocessing for epilepsy monitoring unit patients and dogs

For each subject, if different seizures were recorded at multiple sampling frequencies, all of the recordings were first downsampled to the lowest sampling frequency. Noisy channels were removed based on visual inspection and short missing segments (<0.05 s, with the exception of one 0.514 s segment in patient ‘Study 020’) were linearly interpolated. All channels were re-referenced to a common average reference. Each channel’s time series was then bandpass filtered from 1 to 150 Hz (fourth-order, zero-phase Butterworth filter) and notch filtered (fourth-order, 2 Hz width, zero-phase Butterworth filter) at 60 and 120 Hz (IEEG Portal patients and dogs) or 50, 100, and 150 Hz (UCLH patients).

### Seizure iEEG preprocessing for NeuroVista patients

NeuroVista seizure data were previously notch filtered at 50 Hz during the iEEG acquisition and bandpass filtered (second-order, zero-phase Butterworth filter from 1 to 180 Hz) by Karoly *et al.*^7^ We then removed any electrodes with noisy or intermittent signal from the seizure analysis and re-referenced all iEEG to a common average reference.

The NeuroVista data contain time periods of signal dropouts when the iEEG signal was not recorded. We detected and removed periods of signal dropout by using line length to identify iEEG segments with no signal (i.e. a flat time series with no voltage changes). We defined the line length *L* of a time series as

$$L=\frac{1}{T-1}\overset{T-1}{\underset{i=1}{\sum }}\;|x_{i+1}-x_{i}|$$

where *x_i_* is the *i*th time point in a time series with *T* time points.

For each seizure, time-varying line length was computed for each iEEG channel in sliding windows (1/10 s window, 1/20 s overlap). If a time window had at least 8 out of 16 channels with line length ≤0.5, that time window along with the preceding and following time windows were considered missing data. The next section describes how this missing data were handled when computing seizure time-varying functional connectivity.

### Computing seizure time-varying functional connectivity

The time-varying functional connectivity, defined as coherence in six frequency bands (delta 1–4 Hz, theta 4–8 Hz, alpha 8–13 Hz, beta 13–30 Hz, gamma 30–80 Hz, high gamma 80–150 Hz), was computed for each seizure using a 10 s sliding window with a 9 s overlap between consecutive windows, as in previous work.^4^ Coherence in each frequency band was computed using band-averaged coherence, defined as

$$C_{i,j}=\frac{{\left|\sum_{\;f=f_{1}}^{\;f_{2}}\;P_{i,j}(f)\right|}^{2}}{\sum_{\;f=f_{1}}^{\;f_{2}}\;P_{i,i}(f)\sum_{\;f=f_{1}}^{\;f_{2}}\;P_{\;j,j}(f)}$$

where *f*_1_ and *f*_2_ are the lower and upper bounds of the frequency band, *P*_*i*,*j*_(*f*) is the cross-spectrum density of channels *i* and *j*, and *P*_*i*,*i*_(*f*) and *P*_*j*,*j*_(*f*) are the autospectrum densities of channels *i* and *j*, respectively. For each 10 s window, autospectra and cross-spectra were calculated using Welch’s method (2 s sliding window with 1 s overlap). As noted previously, many seizures in NeuroVista patients contained missing data due to signal dropouts. We tolerated some missing data in this cohort by allowing functional connectivity in each 10 s window to be estimated using a subset of the 2 s Welch subwindows. Specifically, for each functional connectivity time window, we only treated the 10 s functional connectivity time window as missing data if ≥5 of the 2 s subwindows contained missing data. Any NeuroVista seizures with missing functional connectivity time windows were excluded from the remainder of the analysis.

Each resulting coherence matrix was re-expressed in vector form by re-arranging the upper-triangular, off-diagonal elements into a vector of length (*n*^2^ − *n*)/2, where *n* is the number of iEEG channels, and the vector was normalized to have an *L*1 norm of 1. Each seizure time window was therefore represented by a total of 6 × (*n*^2^ − *n*)/2 features that captured the pairwise channel interactions in the six different frequency bands. In a seizure with *m* time windows, the seizure’s time-varying functional connectivity was described by a multivariate time series with 6 × (*n*^2^ − *n*)/2 features and *m* time points.

To reduce noise in the connectivity matrices, patterns of recurring functional connectivity were extracted in each subject using stability non-negative matrix factorization (NMF)^33,34^ using the same pipeline as in our previous work.^4^ The NMF decomposition was used to reconstruct a low-rank approximation of the seizure functional connectivity time series that was used for all downstream analysis. The time-varying functional connectivity of each seizure was also referred to as ‘seizure network evolutions’ and ‘seizure pathways’ throughout the text.

### Visualizing seizure pathways

To visualize changes in seizure networks within and between seizures, each subject’s seizure time-varying functional connectivity (‘seizure pathway’) was projected into two-dimensional space using Sammon mapping, a variation of multidimensional scaling.^35^ The mapping approximated the *L*1 distances between the functional connectivity patterns of each pair of seizure time windows such that seizure time windows with more similar functional connectivity were placed closer together in the projection.

### Comparing seizure pathways using pathway dissimilarities

We used the approach of Schroeder *et al.*^4^ to compare pairs of seizure pathways, which were described by the seizure time-varying functional connectivity, within each subject. Briefly, for each pair of seizures, we used dynamic time warping (DTW)^36^ (MATLAB function *dtw*) to align similar time points in their functional connectivity time series and minimize the overall *L*1 distance between the pair of time series. The seizure pair’s ‘pathway dissimilarity’ (previously called ‘seizure dissimilarity’ in Schroeder *et al.*^4^) was then defined as the average *L*1 distance between their warped time series. Repeating this process for each pair of a subject’s *s* seizures yielded the subject’s pathway dissimilarity matrix, a symmetric *s* × *s* matrix containing all of the pairwise pathway dissimilarities.

As we used information of all frequency bands in comparing pathways, in Supplementary Text 9, we also show the influence of individual frequency bands.

### Comparing seizure durations using duration differences

To compare seizure durations, we computed a pairwise ‘duration difference’ measure for each pair of a subject’s seizures. First, as in previous work,^6^ we transformed each subject’s seizure durations by computing their natural logarithm, which made each subject’s distribution of seizure durations closer to a normal distribution. We then defined the duration difference between a pair of seizures as the absolute difference between their transformed durations,

$$\left|ln(l_{i})-ln(l_{j})\right|$$

where *l*_*i*_ and *l*_*j*_ are the durations, in seconds, of seizures *i* and *j*, respectively. Due to the properties of logarithms, this measure is equal to

$$\left|ln(l_{i}/l_{j})\;\;\right|$$

and therefore depends on the ratio between the durations of seizures *i* and *j*. As such, duration differences capture the proportional differences between seizure durations. For example, the duration difference between a 20 s seizure and a 40 s seizure will be the same as the duration difference between a 60 s seizure and a 120 s seizure because in both cases, the longer seizure is twice the duration of the shorter seizure. Likewise, a certain absolute change in duration, such as 10 s, results in a larger duration difference when the original seizure is shorter. As for the pathway dissimilarity measure, duration differences were computed for each pair of a subject’s *s* seizures to create the subject’s symmetric *s* × *s* duration difference matrix.

### Comparing pathway dissimilarities and duration differences

To compare pathway dissimilarities and duration differences, we used the same approach as Schroeder *et al.*^4^ for comparing dissimilarity matrices. For each subject, we computed Spearman’s correlation between the upper triangular elements of their pathway dissimilarity and duration difference matrices. We then used the Mantel test^37^ (10 000 permutations, one-sided test) to determine the probability of obtaining a correlation greater than or equal to the observed correlation by chance.

### Defining truncated seizures

We found seizure pairs where one seizure is a truncated version of the other both in terms of pathway and duration. To find such ‘truncation pairs’, for each subject we first separated seizures into groups of very similar pathways and found one representative seizure to reduce the later computational cost. This grouping was achieved by performing hierarchical clustering (unweighted pair group method with arithmetic mean [UPGMA]) and cutting the dendrogram at pathway dissimilarity 1. In each group, we then found the centroid seizure, defined as the seizure with the lowest mean pathway dissimilarity to all other seizures in the group, as a representative pathway.

For each pair of a subject’s seizure groups, we first determined if all seizures in one group, labelled Group A, (i) had shorter duration and (ii) had smaller pathways in functional network space than all seizures of the other group, Group B. To define pathway size for each seizure for criteria (ii), we computed the maximum cityblock distance between the functional networks of all pairs of time windows of a seizure’s pathway. If the seizure only had one time window, the maximum distance was zero. If these criteria were met, we then determined whether (iii) pathways in Group A (i.e. the shorter and smaller seizures) were a truncated version of pathways in Group B. For this criteria, we determined if Group A’s representative pathway (‘Pathway A’) had low pathway dissimilarity to the beginning of Group B’s representative pathway (‘Pathway B’). We computed this ‘partial pathway’ dissimilarity between Pathway A and the first *m* time windows of Pathway B, scanning *m* from 1 to the number of time windows in Pathway B. If there existed an *m* for which the partial pathway dissimilarity was ≤1, these seizures were considered a truncation pair, with Pathway A a truncated version of Pathway B. Since these pathways were highly representative of their entire groups, all of seizures in Group A were therefore truncated versions of all seizures in Group B in terms of both their pathways and duration. Note that our implementation of partial matching of pathways is one of many possible versions. Future work may wish to investigate other variations (see e.g. Tormene *et al.*^38^).

After repeating this process for all pairs of seizure groups, a subject’s proportion of truncation pairs was defined as the proportion of their seizure pairs that were related via a truncation.

### Defining elasticity and semblance

To determine the prevalence of elasticity and semblance, we first set thresholds for defining whether a seizure pair had similar pathways (pathway dissimilarity ≤1) and similar durations (duration difference ≤0.2). The pathway threshold was chosen because seizures with pathway dissimilarities below the threshold tend to have visually similar pathways and electrographic patterns. The duration difference threshold allows a *e*^0.2^ = 1.22 fold increase in duration relative to the shorter seizure before the durations are considered different. These thresholds were set so that the overall proportion, across all subjects, of seizure pairs with similar pathways was comparable to the proportion of seizure pairs with similar durations (32.6% and 34.7%, respectively). Seizure pairs with similar pathways (pathway dissimilarity ≤1) and different durations (duration difference >0.2) were then defined as examples of elasticity. Seizure pairs with different pathways (pathway dissimilarity >1) and similar durations (duration difference ≤0.2) were considered examples of semblance. Note that our criteria set here compared entire seizure pathways, and not partial best matches in seizure pathways. Thus, to identify elastic pathways, we required the entire pathways of the two seizures to be similar.

### Comparing duration populations and seizure pathways

In each subject, we determined the number of duration populations by clustering seizure durations (after the natural logarithm transformation) using *k*-means, with the number of clusters *k* scanned from 1 to the subject’s number of seizures, up to a maximum of 10 clusters. The gap statistic^39^ (MATLAB *evalclusters*, search method *firstMaxSE*, with 1000 reference distributions) was used to select the optimal number of clusters, with *k* = 1 indicating an absence of multiple duration populations and *k* ≥ 2 revealing multiple duration populations.

In subjects with multiple duration populations, we additionally clustered seizure pathways into an equivalent number of groups to compare seizure clusters based on duration with clusters based on pathways. In each subject, seizure pathways were clustered by applying UPGMA hierarchical clustering to the pathway dissimilarity matrix, and the resulting dendrogram was then cut to produce the same number of discrete pathway clusters as duration populations. The Rand index and adjusted Rand index (ARI) were then computed to compare the duration population and pathway clusters partitions. To determine the *P-*values of these measures, the cluster membership for one partition was permuted 10 000 times and the measures were recomputed for each permuted partition to create null distributions.

### Correction for multiple comparisons

We report *P* -values for reference throughout the paper. We applied the Benjamini–Hochberg false discovery rate false discovery rate (FDR) correction,^40^ with *α*  = 0.05, to the set of *P*-values from all statistical tests in this work and corresponding Supplementary material. Uncorrected *P*-values are reported in the text, with supplementary results showing statistical significance determined after FDR correction.

### Code and data availability

All analyses were performed using custom code and built-in functions in MATLAB 2018b. NMF was performed using the Nonnegative Matrix Factorization Algorithms Toolbox (https://github.com/kimjingu/nonnegfac-matlab/).^41,42^ The seizure iEEG data of the IEEG Portal EMU patients and dogs are available at www.ieeg.org.^31,32^ The NeuroVista seizure iEEG data used in this study are available from www.epilepsyecosystem.org. The processed data (NMF *W* and *H* matrices) and seizure durations of all subjects, along with analysis code, are available on Zenodo (DOI 10.5281/zenodo.5503590).

## Results

**Figure 1:**
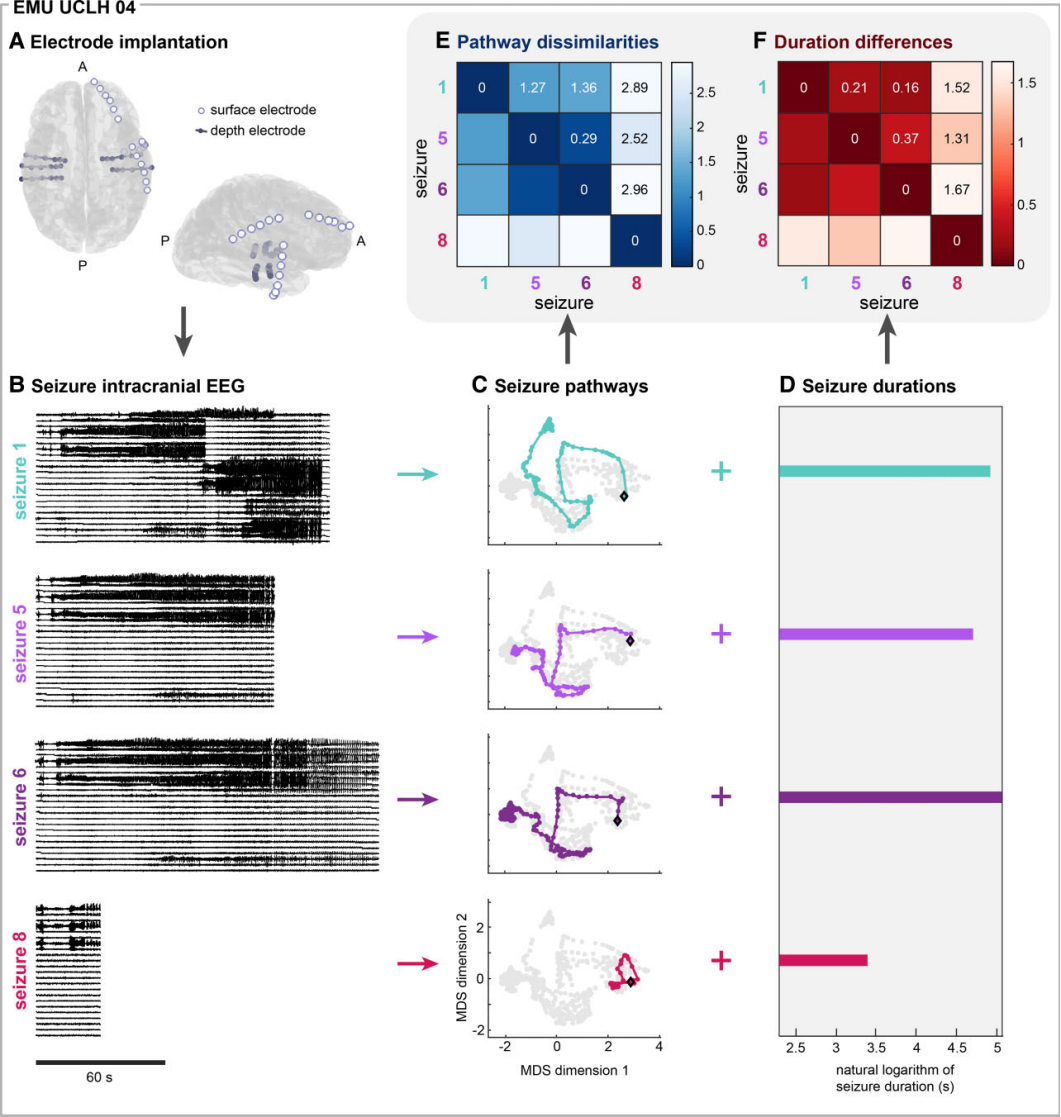
**Quantitatively comparing seizure pathways and durations within individual subjects.** (**A**) Schematic of the electrode implantation for iEEG recording of an example subject, EMU UCLH 04. A = anterior of brain, P = posterior of brain. (**B**) Intracranial EEG of four of EMU UCLH 04’s seizures. The recordings from a representative subset of electrodes are shown. Seizure numbers refer to their chronological order. (**C**) Multidimensional scaling embeddings of the corresponding pathways of the example seizures. Each point corresponds to the functional network configuration of a seizure time window, and time windows with more similar network configurations are located closer together in the embedding. Colored points correspond to time windows that occurred during the example seizure, with the first time window marked with a black diamond and successive time window connected with the colored line to form the seizure pathway. Time windows that occurred during other seizures are shown in grey for reference. (**D**) The durations of each of the example seizures, shown on a natural logarithm scale. Seizure dynamics were characterized by seizure pathways (**C**) and seizure durations (**D**). (**E**) Pairwise pathway dissimilarities and (**F**) duration differences of the example seizures. Both matrices are symmetric.

We analyzed a total of 3224 seizures, recorded using iEEG (Fig. 1A), from

- EMU patients: 31 patients (15 females) with focal epilepsy who underwent presurgical monitoring in EMUs (average 16.5 seizures/patient).
- Neurovista patients: 10 patients (four females) with focal epilepsy who underwent chronic recordings as part of the NeuroVista seizure prediction study^28^ (average 252.6 seizures/patient).
- Dogs: Three dogs with naturally occurring canine epilepsy and focal-onset seizures^29,30^ (average 62.3 seizures/subject).

Figure 1B shows the iEEG recordings of four seizures from an example subject, EMU UCLH 04.

### Quantifying within-subject variability in seizure pathways and seizure durations

We described the dynamics of each seizure using two features:

1. The seizure’s functional network evolution, which can be considered a *pathway* through the space of possible functional network interactions (Fig. 1C).
2. The time it takes the seizure to follow its pathway; i.e. the seizure’s *duration* (Fig. 1D).

For clarity, we will only use the terms *short*/*long* to describe seizure temporal duration and *small*/*large* to describe relative amounts of spatial distances followed by seizure pathways through the functional network space. This functional network space does not directly correspond to anatomical distances in the brain, but rather reflects the similarity of brain activity patterns.

We described seizure pathways through network space by computing the time-varying (sliding window) coherence between pairs of iEEG channels across six frequency bands: delta (1–4 Hz), theta (4–8 Hz), alpha (8–13 Hz), beta (13–30 Hz), gamma (30–80 Hz), and high gamma (80–150 Hz).^4^ To quantify similarities and differences in seizure pathways, we used DTW^36^ to compute pairwise dissimilarities between seizures, resulting in a symmetric ‘seizure dissimilarity’ matrix for each subject^4^ (Fig. 1E). In our case, DTW minimized the overall distance between a pair of seizure pathways by selectively stretching parts of each pathway such that similar network configurations were temporally aligned. Therefore, DTW allowed us to recognize similar seizure pathways even if the seizures had different durations. We defined the ‘pathway dissimilarity’ between a pair of seizures as the average distance between their functional connectivity time series after DTW. Additionally, to visualize seizure pathways through network space, we used multidimensional scaling to project each subject’s seizure network evolutions into a two-dimensional space (Fig. 1C, also see the ‘Methods’ section). Each point in the projection corresponds to a network configuration that occurred during the seizure, and time points with more similar network configurations tend to be placed closer together.

To compare variability in seizure pathways to variability in seizure durations, we quantified the pairwise differences in each subject’s seizure durations. As in previous work,^6^ we first computed the natural logarithm of each seizure duration (Fig. 1D). We then computed the pairwise absolute differences between the transformed seizure durations, resulting in a symmetric ‘duration difference’ matrix for each subject (Fig. 1F). Due to the properties of logarithms, our measure captures relative changes in duration (‘Comparing seizure durations using duration differences’ in the ‘Methods’ section). We also observed a wide range of seizure durations in most subjects, and demonstrate additional reasons to use log distances (Supplementary Text 2).

Thus, each subject’s spatiotemporal seizure variability was described by two matrices: a pathway dissimilarity matrix (Fig. 1E), containing pairwise comparisons of seizure pathways through network space, and a duration difference matrix (Fig. 1F), composed of pairwise differences of seizure durations. In our subsequent analyses, we used these two measures to explore the relationship between seizure pathways and seizure durations in each subject. As such, our analysis focused on *differences* in seizure pathways and durations between pairs of seizures, rather than the pathway and duration features themselves. This seizure pair approach had two main advantages. First, unlike seizure duration, seizure pathways do not map onto a single feature that changes from seizure to seizure.^4^ However, our pairwise measures allowed us to ask questions such as, ‘Does a pair of seizures have similar pathways if and only if they have similar durations?’ Second, comparing these features at the seizure pair level was a more appropriate analysis for features that vary on a spectrum. In many subjects, seizures cannot be clearly grouped based on their pathways^4^ or durations^6,7^ because these features vary continuously, producing a spectrum of seizure dynamics. Thus, our pairwise approach allowed us to precisely compare seizure pathways and durations in all subjects.

### The strength of the relationship between seizure pathways and seizure durations varies across subjects

**Figure 2:**
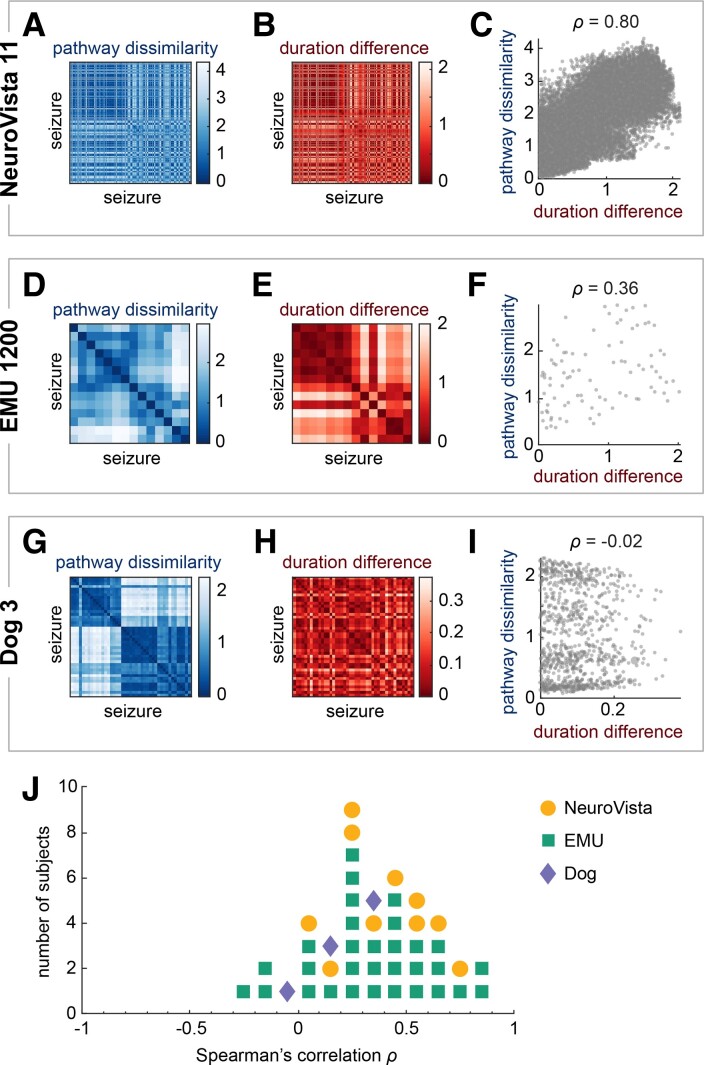
**Comparison of pathway dissimilarities and duration differences**. (**A–I**) Comparison of pathway dissimilarities and duration differences in three example subjects: NeuroVista 11 (351 seizures), EMU UCLH 17 (14 seizures), and Dog 3 (43 seizures). (**A, D and G**) Pathway dissimilarity matrices of the example subjects. Each matrix quantifies the pairwise dissimilarities of the subject’s seizure pathways. (**B, E and H**) Duration difference matrices in the same subjects. Each matrix quantifies the pairwise differences in the subject’s seizure durations on a natural logarithm scale. (**C, F and I**) Scatter plots and Spearman’s correlations of each subject’s pathway dissimilarities versus duration differences. Each point corresponds to a seizure pair. (**J**) Dot plot of the Spearman’s correlations between pathway dissimilarities and duration differences of all subjects. Each marker corresponds to a subject, with the color and shape indicating the subject’s cohort.

We first compared each subject’s pathway dissimilarity matrix to their duration difference matrix. Figure 2 shows the matrices of three example subjects, one from each cohort. Visually and quantitatively comparing the matrices within each subject revealed that their concordance varied across subjects. NeuroVista 11’s pathway dissimilarity (Fig. 2A) and duration difference (Fig. 2B) matrices had very similar structures, indicating that seizures with similar (dissimilar) pathways also had similar (different) durations. The Spearman’s correlation between these two matrices was, as expected, very high (Fig. 2C, *ρ*  = 0.80). On the contrary, Dog 3’s matrices (Fig. 2G, H and I, *ρ*  = −0.02) had different structures, suggesting little or no relationship between pathway dissimilarity and duration differences. EMU UCLH 17’s matrices (Fig. 2D, E and F, *ρ*  = 0.36) were between these two extremes: while there were some similarities across the two matrices, each matrix also had distinct patterns. These examples demonstrate that the relationship between seizure pathways and seizure durations differed across subjects.

In most subjects, pathway dissimilarities and duration differences were weakly to moderately correlated (Fig. 2J, median correlation: 0.322, first quartile: 0.191, third quartile: 0.537). Supplementary Text 4 provides additional information on the statistical significance of these associations for reference. Supplementary Text 5 shows that the association strength is also not determined by the range in either feature, indicating that the level of variability in pathways or duration did not influence their relationship. The weak to moderate correlations revealed that changes in seizure durations were not fully explained by changes in seizure pathways and *vice versa*. Therefore, seizure pathways and durations contained complementary information about the dynamics of a given seizure.

### The relationship between pathways and durations is strengthened by concordant and truncated pathways and durations

We next examined how pairwise relationships between seizures could strengthen or weaken the association between seizure pathways and seizure duration within each subject. A pair of seizures could fall into one of four possible categories:

1. The seizure pair had similar pathways and similar durations.
2. The seizure pair had different pathways and different durations.
3. The seizure pair had similar pathways, but different durations.
4. The seizure pair had different pathways, but similar durations.

**Figure 3:**
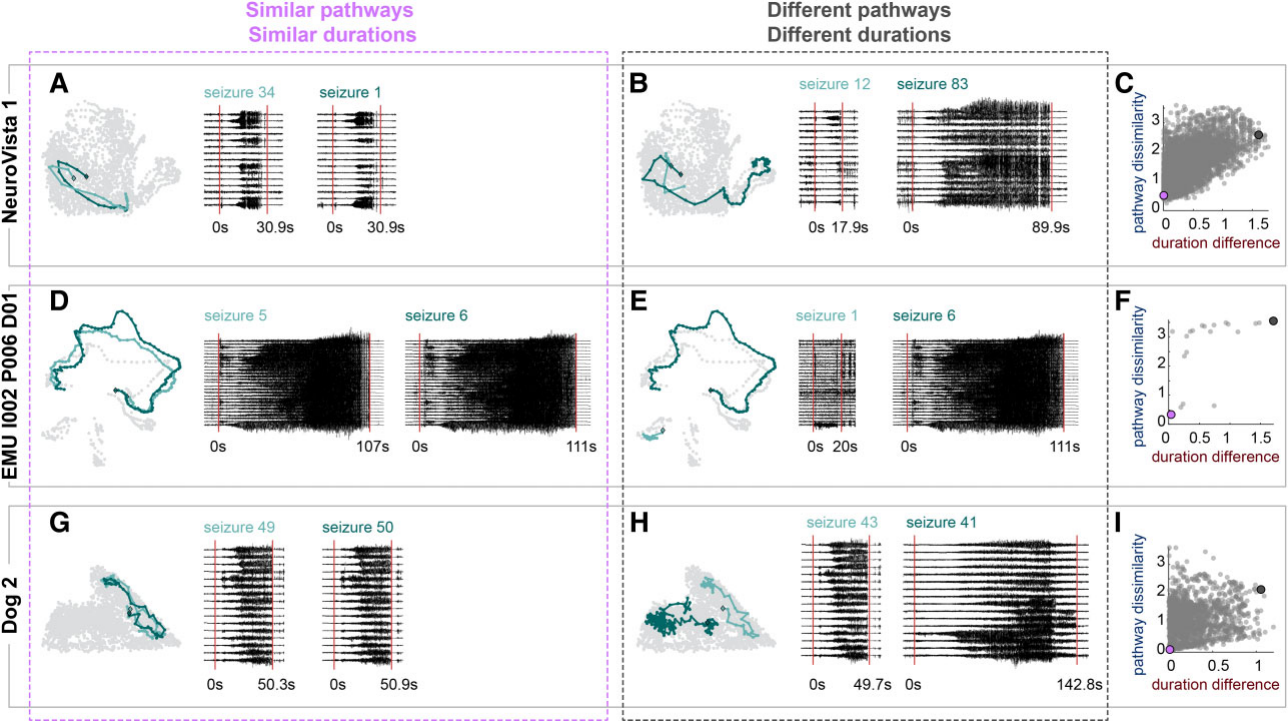
**Example seizure pairs that strengthen the relationship between seizure pathways and seizure durations**. (**A, D and G**) Example pairs of seizures with similar pathways and similar durations. Left: the seizures’ pathways (light teal and dark teal), embedded in network space as in Fig. 1C. The time points in the subjects other seizure pathways are shown for reference (light grey points). Right: The iEEG traces and durations of each pair of seizures, with 10 s of preictal and postictal data also shown. Red vertical lines mark seizure onset and termination. For EMU I002 P006 D01, a representative subset of channels is shown. (**B, E and H**) For the same subjects as in **A, D, and G**, example pairs of seizures with different pathways and different durations. Visualization formats are the same as in **A, D, and G**. For **A, B, D, E, G, and H**, the time and voltage scales of the iEEG traces are consistent for each subject, but not across subjects. (**C, F and I**) Scatter plots of pathway dissimilarities versus duration differences of the three example subjects, with the example seizure pairs highlighted with large purple points (similar pathways, similar durations) and large dark grey points (different pathways, different durations).

We initially evaluated cases in which the seizure pair’s pathway and duration agreement was concordant (i.e. both features similar or both features different, Cases 1 and 2). Figure 3 shows example pairs of seizures that had similar pathways and similar durations (Case 1, Fig. 3A, D  and G) or different pathways and different durations (Case 2, Fig. 3B, E  and H). In the latter case, pathways could either partially overlap in network space (Fig. 3B) or occupy distinct regions (Fig. 3E and H). Therefore, these disparate pathways could either share network features or have completely unrelated evolutions.

Figure 3C, F  and I visualizes how these pairs of seizures impact the relationship between pathway and duration variability in each of the example subjects. When pathways and durations were similar, the seizure pair had a low pathway dissimilarity and a low duration difference (purple points). In contrast, pairs of seizures with different pathways and durations had high pathway dissimilarities and high duration differences (dark grey points). The combination of such seizure pairs within the same subject contributed to the positive correlations between pathway dissimilarities and duration differences.

**Figure 4:**
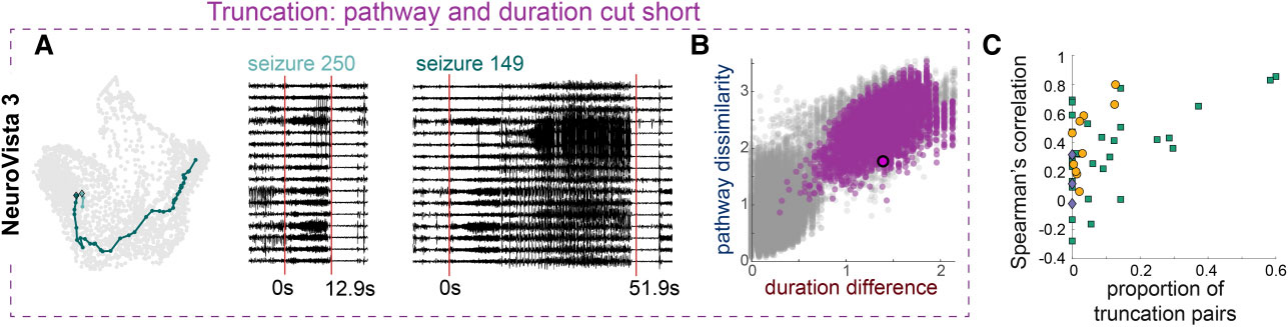
**Truncating seizures in both pathway and duration strengthens the relationship between pathways and duration**. (**A**) Example pathways of a pair of seizures, where one is a truncated version of the other, in NeuroVista 3. The corresponding iEEG traces and durations of the pair of seizures, with 10 s of preictal and postictal data also shown. Red lines mark seizure onset and termination. (**B**) Scatter plot of pathway dissimilarities versus duration differences NeuroVista 3, with the example seizure pairs highlighted in purple with black outline. Other purple markers show all other truncated pairs of seizures in this subject. Grey markers show all other seizure pairs. (**C**) Scatter plot of the Spearman’s correlation between pathway dissimilarity and duration differences in each subject against the proportion of truncation pairs in that subject. Marker conventions as in Fig. 2J. Subjects with a higher proportion of truncation pairs tended to have a higher Spearman’s correlation between pathway dissimilarities and duration differences (*ρ*  = 0.42, *p* = 0.005).

Coinciding changes in both seizure pathways and seizure durations also strengthened this relationship. A special case is given by a seizure pair, where one seizure is a truncated version of the other seizure in both pathway and duration. We termed such seizure pairs ‘truncation pairs.’ Figure 4A shows an example truncation pair in Neurovista 3, and Fig. 4B demonstrates that this seizure pair contributed to a positive correlation between pathway dissimilarities and duration differences. Indeed, all truncation pairs had this effect (Fig. 4B, purple markers) and contributed to a high Spearman’s correlation (*ρ*  = 0.66) in this subject. Finally, we assessed if truncation pairs contributed to a stronger relationship between pathways and duration across all subjects. Figure 4C supports this hypothesis and demonstrates that in general, subjects with a larger proportion of truncation pairs had a higher correlation between pathway dissimilarities and duration differences. In other words, pathway and duration variability were related when changes in pathways produced concordant changes in durations and *vice versa*, and pathway and duration truncation is one mechanism that produces such concordant changes.

### The relationship between pathways and durations is weakened by elasticity and semblance

**Figure 5:**
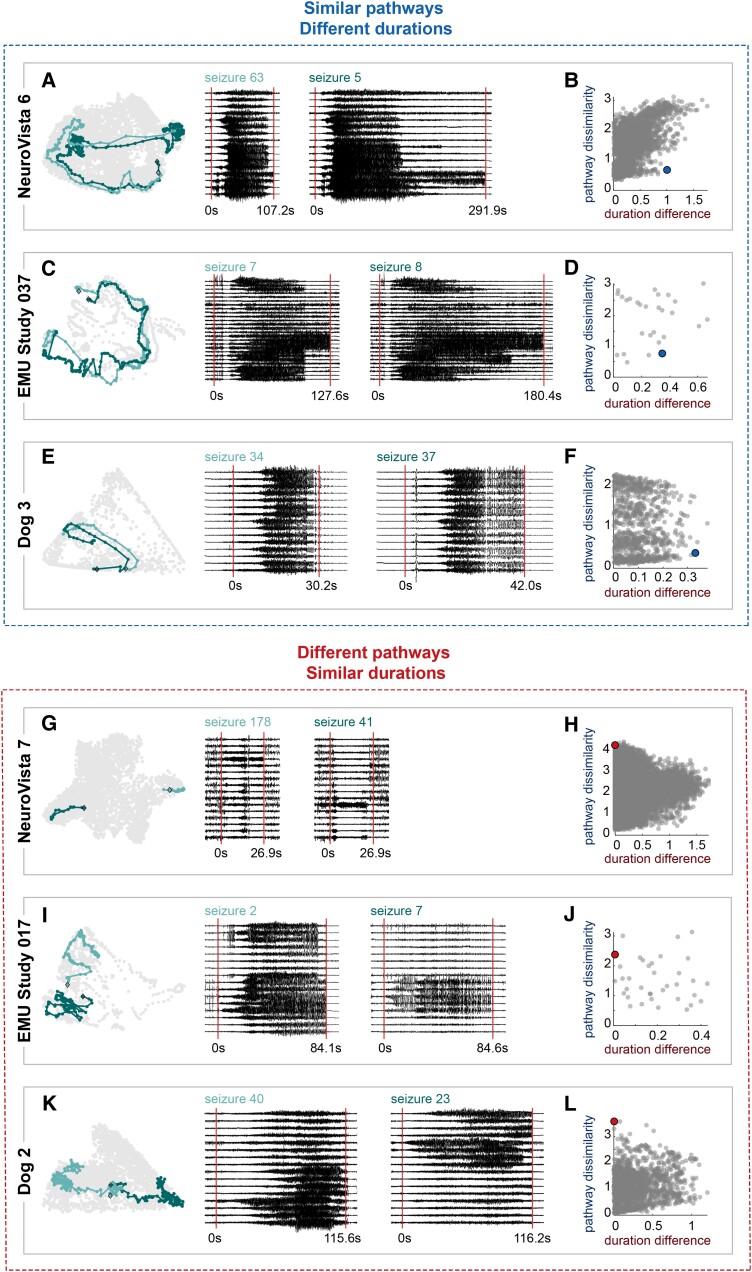
**Example seizure pairs that weaken the relationship between seizure pathways and seizure durations**. (**A–F**) Examples of seizure pairs with similar pathways and different durations (‘elasticity’). (**A, C and E**) Visualization of the seizure pathways, durations and iEEG is the same as in Fig. 3. For EMU Study 037, a representative subset of channels is shown. (**B, D and F**) Scatter plots of pathway dissimilarities versus duration differences for each subject, with the example seizure pairs highlighted with larger blue points. (**G–L**) Examples of seizure pairs with different pathways and similar durations (‘semblance’). (**G**, **I**, **K**) Visualization of the seizure pathways, durations and iEEG, as in Fig. 3H, J and L) Scatter plots of pathway dissimilarities versus duration differences for each subject, with the example seizure pairs highlighted with larger red points.

We next examined how pairs of seizures could weaken the relationship between seizure pathways and durations. First, a pair of seizures could share similar pathways for the entirety of the seizure, but have different durations (Case 3). Figure 5A–F provides three examples of this scenario. Although the seizures in each pair followed similar routes through network space, they took different amounts of time to do so, revealing ‘elasticity’ in time for each example seizure pathway.^26^ Interestingly, the pathways were not uniformly elastic; instead, there appeared to be pathway-specific locations where a pathway dwelled for different amounts of time. For example, in NeuroVista 6, Seizure 5 spent relatively more time in the middle and end of the pathway (Fig. 5A). Due to their shared pathways and different durations, such pairs weakened the relationship between pathways and durations (Fig. 5B, D  and F blue points). These results revealed that a seizure’s duration is not rigidly constrained by its pathway.

The final scenario was that two seizures had different pathways, but the same duration (Case 4). Figure 5G–L illustrates this case, which we termed ‘semblance’ to highlight that two seizures can have the same duration despite different pathways. Thus, the duration of a seizure does not necessarily provide information about a seizure’s pathway. The pairs of seizures in our examples all had low duration differences and high pathway dissimilarities (Fig. 5H, J  and L), again weakening the relationship between pathway and duration variability in each of these subjects.

To determine the prevalence of elasticity and semblance, we set thresholds for whether two seizures had similar pathways and/or similar durations (see the ‘Methods’ section). Almost all subjects displayed elasticity (30/31 EMU, 10/10 NeuroVista, and 3/3 dogs) and semblance (27/31 EMU, 10/10 NeuroVista, and 3/3 dogs) (Supplementary Text 6). Therefore, these mechanisms for independent variability in pathways and durations were widespread in our cohorts.

### Populations of short and long seizures do not reliably correspond to different seizure pathways

In the previous sections, we analyzed the relationship between seizure pathways and seizure durations in all subjects, regardless of the nature of their seizure dynamics. It is possible that pathways and durations are more closely related in subjects whose seizures can be grouped into distinct duration populations of short and long seizures. In particular, previous studies have hypothesized that duration populations correspond to different seizure pathways.^6,7^

**Figure 6:**
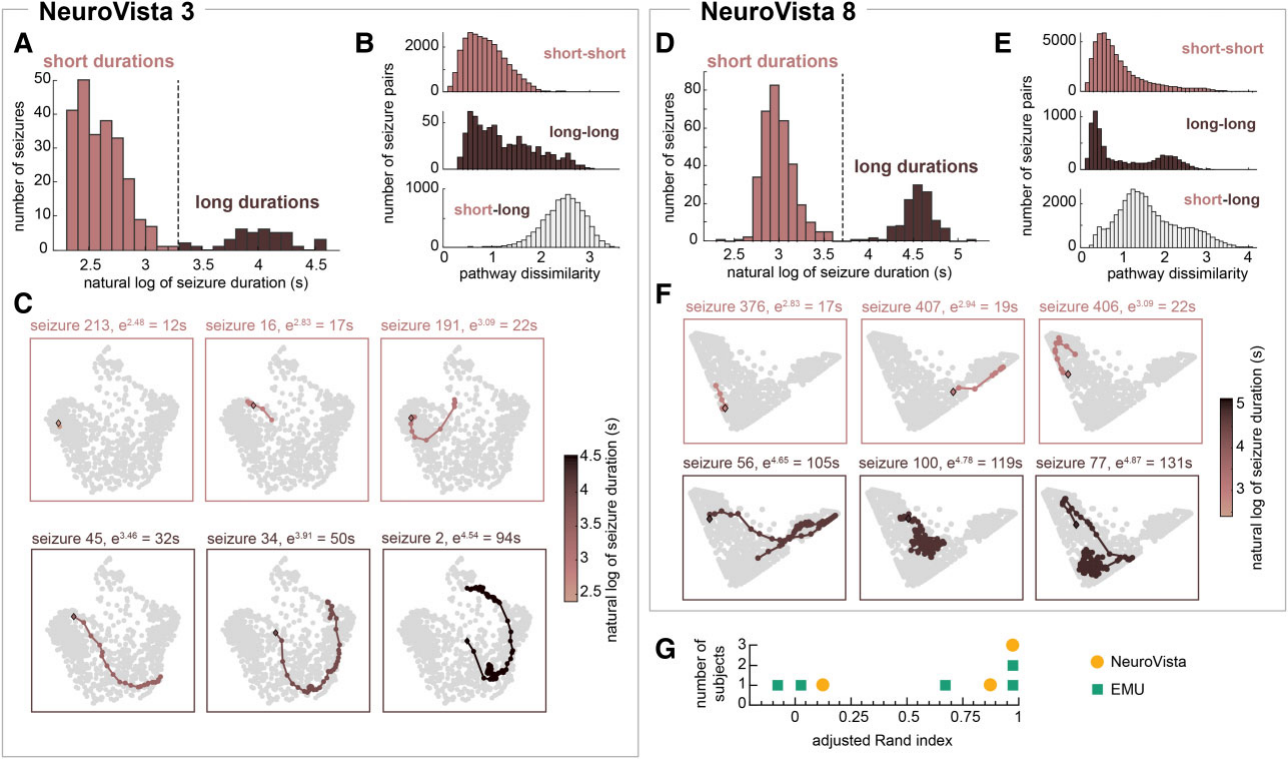
**Short and long seizures do not necessarily correspond to different seizure pathways.** Analysis of seizure pathways within subjects with multiple duration populations. (**A and D**) Distribution of seizure durations in example subjects NeuroVista 3 (**A**) and NeuroVista 8 (**D**). Each bimodal distribution can be divided into two duration populations: one with short seizures and one with long seizures. (**B and E**) The distributions of pathway dissimilarities between short-short (top), long-long (middle), and short-long (bottom) pairs of seizures in each example subject. (**C and F**) Example seizure pathways of short and long seizures in each subject. (**G**) The ARI between seizure duration populations and seizure pathway clusters in all subjects with duration populations.

As in previous work,^6,7^ we clustered seizure durations in each subject and found those subjects with multiple groups, or populations, of seizures based on their seizure durations. While most subjects did not have multiple duration populations, a total of eight subjects (5/31 EMU patients, 3/10 Neurovista patients, and 0/3 dogs) had two duration populations. Figure 6A–F explores the relationship between these duration populations and the corresponding seizure pathways in two example subjects, NeuroVista 3 and NeuroVista 8. In NeuroVista 3, pairs of seizures tended to have similar pathways if and only if they belonged to the same duration population (i.e. if they were both short or both long) (Fig. 6B and C). Although there was still some pathway variability within each duration population, especially among the long seizures, overall the different duration populations corresponded to different seizure pathways. In contrast, in NeuroVista 8, pairs of seizures with different durations often had more similar pathways than pairs of seizures with similar durations (Fig. 6E). Seizures with similar durations could occupy different parts of network space, while seizures with different durations (e.g. short seizure 407 and long seizure 56) could partially overlap in network space (Fig. 6F). As a result, seizure duration populations did not distinguish different seizure pathways in NeuroVista 8.

To quantify the agreement between seizure pathways and durations populations, we also clustered seizures into two groups based on seizure dissimilarities. We compared these pathway group assignments to duration populations using the ARI (Fig. 6G). An ARI of one indicated perfect agreement between the two partitions, while an ARI close to zero corresponded to only chance levels of agreement. NeuroVista 3 was one of three subjects with an ARI of one, indicating that short and long seizures perfectly corresponded to the division of seizure pathways. Meanwhile, NeuroVista 8’s ARI was only 0.14; in this subject and two others, short and long seizures were not proxies for different seizure pathways. The remaining two subjects had intermediate levels of agreement between pathway groups and duration populations. Supplementary Text 7 contains additional clustering analyses. These results revealed a complex, subject-specific relationship between seizure durations and pathways in subjects with multiple duration populations. In some subjects, duration populations indeed corresponded to different seizure pathways, although there was additional pathway variability within each duration population. In others, duration populations were not associated with different groups of seizure pathways.

### Relationship to other clinical variables

Finally, we investigated whether seizure pathway variability, seizure duration variability, or their relationship was associated with clinical variables, such as disease duration or lobe of epilepsy, in Supplementary Text 8. We could confirm some previously reported relationships with seizure duration, but found no other strong relationships. Specifically, the subject-specific relationship between seizure pathways and durations was not explained by our clinical variables.

## Discussion

We quantitatively compared two seizure features: their duration and their pathway. We found that these features often varied independently within individual subjects: seizures with the same pathway could have different durations, and seizures with the same duration could have different pathways. The level of association between pathways and durations was subject-specific, and we identified multiple mechanisms such as truncating pathways and temporal elasticity that could strengthen or weaken this relationship. Additionally, we found that distinct populations of short and long seizures did not necessarily correspond to different groups of seizure pathways. Thus, seizure pathways and durations carry complementary information about seizures, and these features can perhaps be modulated independently within a given subject. Additionally, the highly subject-specific relationship between seizure pathways and durations highlights the need for statistical and dynamical models that can be tailored to individual data.

The fact that an individual seizure pathway does not have a rigidly predetermined duration indicates that a seizure’s evolution itself does not fully dictate its rate of progression. Thus, a seizure is not a pre-programmed sequence of pathological electrographic patterns with set timings. Rather, both the seizure’s pattern of activity and the timings of those different patterns can change from one seizure to the next. These observations imply that there are factors that modulate the timings within seizure pathways. Our work indicates that these modulators can impact seizure pathways independently of the seizure duration, suggesting that there are multiple biological mechanisms that influence seizure features. Identifying these mechanisms could offer therapeutic targets for controlling seizures.

Based on our observation of temporally elastic seizure pathways, one potential therapeutic approach would be to reduce the duration of a given seizure pathway. To achieve this goal, further work is needed to determine the biological mechanisms that produce changes in seizure duration among seizures with similar pathways. Consistent with our observations, Wenzel *et al.*^26^ previously observed seizures with similar propagation patterns, but different durations, in a rodent model. They described this feature as ‘elasticity’ of seizure propagation. To our knowledge, seizure elasticity has not been previously quantitatively described in humans, although seizures with consistent neuronal spiking patterns, but different durations, have been observed.^27^ Our work reveals that temporal elasticity is also a common feature of human seizures. Interestingly, it appears that elasticity does not necessarily affect the entire seizure pathway; instead, a seizure could selectively dwell in certain parts of a given pathway. Further research is needed to understand what parts of seizure pathways are most prone to variable rates of progression as well as the underlying molecular mechanisms, such as local^26^ or feedforward^43^ inhibition, that determine these temporal features. Uncovering these mechanisms could provide possible clinical strategies for controlling seizure progression and duration, thus reducing seizure severity.

We also identified a mechanism that can shorten both seizure pathways and durations, which we termed ‘truncating’ a seizure pathway. Thus, it is likely that some seizure modulators affect both seizure pathways and durations, whether by truncations or other mechanisms. Using the same chronic patient recordings as our work, Karoly *et al*.’s^7^ earlier modelling study also found evidence that shorter seizures may be created by early terminations along a given seizure pathway. Specifically, they observed that variability in a patient’s seizure duration was associated with seizure terminations, but not onsets.^7^ While this work used a different computational approach to characterize seizure pathways, these results suggest that seizures with different durations can share the same initial evolution. Additionally, microelectrode recordings have revealed that some patients have shorter seizures that terminate earlier along the patient’s characteristic seizure evolution, again revealing that shorter seizures can arise by truncating seizure pathways.^44^ Biological triggers of this truncation mechanism could potentially be used as a clinical approach to induce early termination of seizure pathways and thereby reduce seizure duration and severity.

Our cohort included subjects with a spectrum of seizure durations as well as subjects with distinct populations of short and long seizures. Past studies uncovered the presence of such duration populations in the NeuroVista cohort that was analyzed in this study.^6,7^ Interestingly, we observed that populations of short and long seizures only corresponded to different groups of seizure pathways in some subjects, such as NeuroVista 3. These subjects likely underlie previous evidence for a link between duration populations and different seizure pathways.^7^ Mechanisms such as truncating pathways could potentially create such duration populations.^7^ However, in other subjects, such as NeuroVista 8, we found that a seizure’s duration population was not closely linked to its pathway; indeed, short and long seizures could have more similar pathways to each other than to seizures with similar durations. It is possible that distinct duration populations arise via different mechanisms in such cases. Further research is needed to determine whether the relationship between seizure pathways and duration populations impacts seizure forecasting^6^ and seizure duration predictions^45^ in these patients.

Clinical factors such as seizure localization are known to impact seizure durations across patients.^17,46^ However, the factors that modulate seizure durations within the same patient are unknown. We previously hypothesized that preictal variability in brain dynamics^4^ or continuous fluctuations in interictal brain dynamics^47^ could produce changes in seizure pathways. Likewise, fluctuations in interictal markers^48^ such as levels of cortical excitability and inhibition,^49–51^ interictal spike rate,^52,53^ or functional networks^54^ could potentially affect seizure duration. Additional factors such as sleep state^55^ and temporal seizure clusters^17^ are known to impact seizure duration, but it is unclear whether these duration changes occur as a byproduct of coinciding changes in seizure pathways. Indeed, in a rodent model, seizure durations, severity, and spread all change over the course of a seizure cluster,^56^ suggesting that some mechanisms influence both seizure pathways and durations. Disentangling the factors that shape seizure pathways and durations will require accounting for variability in one feature when analyzing the other aspect of seizure dynamics. Indeed, our additional analysis of the NeuroVista patients suggests that some underlying factors solely shape seizure pathways, while others determine the dwell time in specific parts of the pathway.^57^ Additionally, this separate analysis suggests that these seizure features change over circadian and multidien timescales, potentially due to modulatory factors with the same temporal fluctuations.^49,58^

One promising avenue to understanding seizure pathways and their durations is developing computational models that capture not only specific stages of a seizure, but also neural dynamics throughout the seizure’s evolution. Many studies have focused on computationally analyzing^8,9,59–62^ or modelling^5,13,63,64^ variability in seizure onset and termination dynamics. For example, Jirsa *et al.*^13^ characterized seizures by the types of dynamic transitions that occurred at seizure start and end. Using this approach, Saggio *et al.*^5^ classified seizures into ‘dynamotypes’ and uncovered within-patient variability in these classes. While their classification characterizes seizure onset and termination, Saggio *et al.*^5^ also developed a model that explains relationships between dynamotypes as well as more complex seizure dynamics such as status epilepticus. Such models can therefore also capture the seizure’s full evolution, or dynamical pathway.^7,12^ Thus, computational models of seizure evolutions could be extended to explore the dynamical mechanisms underlying other types of seizure variability beyond seizure transitions, such as the variability in seizure pathways, dwell sites, and overall duration that we observed in this work.

Our study was limited to human patients with drug-resistant focal epilepsies and dogs with focal-onset seizures, and it is unclear whether similar relationships between seizure pathways and durations exist in other types of epilepsies. Our concordant findings in dogs indicate that our results generalize beyond human patients. It is also likely that we did not observe all types and combinations of pathway and duration variability in our subjects, especially in EMU patients with shorter recordings.^65^ Another limitation of our study is that seizure duration depends on clinically or algorithmically marked seizure onsets and terminations. Clinical markings can be subjective and vary from marker to marker, especially in some seizures with more ambiguous onsets.^14,66^ However, marking errors were likely small and non-systematic relative to the length of most seizures.

We have shown that seizure pathways and durations can vary independently within the same patient, increasing the possible combinations of seizure dynamics that can occur in a given patient. As such, both pathway and duration information is needed to fully characterize a seizure. Determining the mechanisms by which each feature independently varies and co-varies could lead to strategies for reducing seizure duration and severity in therapeutic interventions.

## Supplementary Material

fcac173_Supplementary_Data

## Abbreviations

ARI =: adjusted Rand index
DTW =: dynamic time warping
EMU =: epilepsy monitoring unit
FDR =: false discovery rate
iEEG =: intracranial EEG
NMF =: non-negative matrix factorization
UCLH =: University College London Hospital
UPGMA =: unweighted pair group method with arithmetic mean
