# Supplementary material for: Multiple mechanisms shape the relationship between pathway and duration of focal seizures
Source: Brain Commun. 2022 Jul 6;4(4):fcac173. doi: 10.1093/braincomms/fcac173 (PMC9280328; doi:10.1093/braincomms/fcac173)
Supplement: fcac173_Supplementary_Data [file fcac173_supplementary_data.pdf]

Supplementary for: Multiple mechanisms shape the relationship between pathway and duration of focal seizures

Gabrielle M. Schroeder[1], Fahmida A. Chowdhury[3], Mark J. Cook[4,6], Beate Diehl[3], John S. Duncan[3], Philippa J. Karoly[4,5,6], Peter N. Taylor[1,2,3], Yujiang Wang[1,2,3*]

June 9, 2022

1. CNNP Lab (www.cnnp-lab.com), Interdisciplinary Computing and Complex BioSystems Group, School of Computing, Newcastle University, Newcastle upon Tyne, United Kingdom

2. Faculty of Medical Sciences, Newcastle University, Newcastle upon Tyne, United Kingdom

3. UCL Queen Square Institute of Neurology, Queen Square, London, United Kingdom

4. Graeme Clark Institute and St Vincent's Hospital, University of Melbourne, Melbourne, Victoria, Australia

5. Department of Biomedical Engineering, University of Melbourne, Melbourne, Victoria, Australia

6. Seer Medical Pty Ltd, Melbourne, VIC, Australia

* Contact Dr Yujiang Wang

School of Computing, Newcastle University,

NE4 5TG, Newcastle upon Tyne,

Yujiang.Wang@newcastle.ac.uk

Supplementary Materials

Supplementary Text 1 Subject metadata

Table 1 provides the following metadata for the epilepsy monitoring unit (EMU) patients:

- **Hospital:** hospital at which the patient underwent presurgical monitoring.
- **Age:** age, in years, at the time of the presurgical monitoring.
- **Sex:** patient sex.
- **Hemisphere:** purported hemisphere of onset of the patient's seizures, based on clinical findings.
- **Lobe:** purported lobe of onset of the patient's seizures, based on clinical findings. Note that some patients had seizures arising from multiple lobes/at the boundary of two lobes (e.g., OP = occipital/parietal onset).
- **Pathology:** postoperative tissue pathology findings.
- **ILAE surgical outcome:** patient surgical outcome according to the International League Against Epilepsy classification (1 = seizure free, 2 = only auras, 3+ = not seizure free). A dash indicates that the patient did not undergo surgery or their surgical outcome is unavailable. For IEEG Portal patients (MC and HUP hospitals), the surgical outcome provided by the database is given. For University College London Hospital (UCLH) patients, the 12 months post-surgical outcome is provided.
- **Total recording time:** total duration of the presurgical intracranial recording time.
- **# seizures analysed:** number of the patient's seizures analysed in this work.
- **# electrodes analysed:** number of recording electrodes included in the analysis, after removing noisy electrodes.
- **Sampling frequencies:** sampling frequencies at which intracranial data was acquired and stored.

- **AED reduction performed:** whether patient antiepileptic drug (AED)s were systematically reduced during the presurgical recording. A dash indicates that this information is unavailable.

Table 2 provides the following metadata for the NeuroVista patients:

- **Age (yrs):** patient age in years.
- **Sex:** patient sex.
- **Age at diagnosis (yrs):** patient age when they were diagnosed with epilepsy, in years.
- **Lobe:** purported lobe of onset of the patient's seizures, based on clinical findings. Note that some patients had seizures arising from multiple lobes (e.g., OP = occipital/parietal onset).
- **Previous resection**: whether the patient had undergone surgical resection prior to the chronic recording.
- **# seizures analysed:** number of the patient's seizures analysed in this work.
- **# electrodes analysed:** number of recording electrodes included in the analysis after removing noisy electrodes.
- **Total recording time (days):** total duration of the intracranial recording time, in days.
- **Sampling frequency:** sampling frequency at which intracranial data was acquired and stored.

Table 3 provides the following metadata for the dogs:

- **Total recording time:** total duration of the intracranial EEG (iEEG) recording.
- **# seizures analysed:** number of the subjects's seizures analysed in this work.
- **# electrodes analysed:** number of recording electrodes included in the analysis, after removing noisy electrodes.
- **Sampling frequencies:** sampling frequencies at which intracranial data was acquired and stored.

Subject	Hospital	Age (yrs)	Sex	Hemisphere	Lobe	Pathology	ILAE surgical outcome	Total recording time	# seizures analysed	# electrodes analysed	Sampling frequencies	AED reduction performed?
**Study 012-2**	MC	37	M	B	T	Other	-	13d 16h	28	81	499.907 Hz	-
**Study 017**	MC	39	M	R	FT	Other	4	7d 17h	9	16	499.907 Hz	-
**Study 019**	MC	33	M	L	T	-	5	5d 16h	33	96	499.907 Hz	-
**Study 020**	MC	10	M	R	F	-	4	5d	8	55	499.907 Hz	-
**Study 021**	MC	16	M	R	FT	Other	1	6d 11h	13	108	500 Hz	-
**Study 024**	MC	23	F	B	TP, IH	-	-	8d 10h	12	83	500 Hz	-
**Study 026**	MC	9	M	L	F	FCD	1	3d 3h	21	81	499.907 Hz	-
**Study 027**	MC	34	F	L	T	HS	-	3d 21h	6	47	500 Hz	-
**Study 030**	MC	18	F	L	FP	FCD	3	5d 23h	8	63	500 Hz	-
**Study 033**	MC	3	M	L	F	TS	5	6d 17h	17	127	500 Hz	-
**Study 037**	MC	62	F	R	F	-	-	8d 23h	8	78	499.907 Hz	-
**Study 038**	MC	58	M	L	FT	-	1	3d	10	86	500 Hz	-
**I002 P006 D01**	HUP	26	F	R	T	-	-	12d 22h	7	83	512 Hz	yes
**UCLH 01**	UCLH	35	M	L	OP	Other	4	7d 1h	13	56	512 Hz, 1024 Hz	no
**UCLH 02**	UCLH	38	F	B	T	Other	3	6d 19h	6	20	1024 Hz	yes
**UCLH 03**	UCLH	25	F	L	P	FCD	3	4d 3.6h	8	71	512 Hz	no
**UCLH 04**	UCLH	25	F	L	T	HS, BDI	1	6d 4h	9	46	512 Hz	yes
**UCLH 05**	UCLH	28	M	L	T	HS	4	7d	11	58	512 Hz	yes
**UCLH 06**	UCLH	28	F	R	OP	TS	1	1d 19h	40	76	512 Hz	no
**UCLH 07**	UCLH	28	M	L	F	FCD	1	12d 5h	26	73	512 Hz	yes
**UCLH 08**	UCLH	21	F	R	T	HS	2	8d 21h	15	85	512 Hz	yes
**UCLH 09**	UCLH	28	M	L	F	GL	1	1d 20h	8	84	512 Hz	no
**UCLH 10**	UCLH	31	F	R	T	CAV	1	6d 1h	13	53	1024 Hz	yes
**UCLH 11**	UCLH	43	F	R	TOP	DNT	1	7d 22h	24	62	512 Hz, 1024 Hz	no
**UCLH 12**	UCLH	27	F	L	F	FCD	1	8d	8	111	512 Hz	yes
**UCLH 13**	UCLH	39	M	L	P	CAV	4	5d 22h	43	51	1024 Hz	no
**UCLH 14**	UCLH	60	F	L	F	FCD	2	2d	10	94	512 Hz	no
**UCLH 15**	UCLH	28	M	R	P	FCD	3	5d 5h	52	75	512 Hz	no
**UCLH 16**	UCLH	41	M	R	T	HS	3	15d 22h	11	34	1024 Hz	yes
**UCLH 17**	UCLH	24	F	R	T	HS	1	2d 19h	14	71	512 Hz	yes
**UCLH 18**	UCLH	26	M	R	T	Other	3	5d 7h	20	77	512 Hz	yes

Hospitals
MC = Mayo Clinic
HUP = Hospital of the University of Pennsylvania
UCLH = University College London Hospital

Sex
M = male, F = female

Hemisphere
L = left, R = right, B = bilateral

Lobe
T = temporal, F = frontal,
P = parietal, O = occipital,
IH = interhemispheric

Pathology
FCD = Focal cortical dysplasia, BDI = Brain damage - inflammatory,
HS = Hippocampal sclerosis, TS= Tuberous sclerosis, GL = Glioma,
CAV = Cavernoma, DNT = Dysembryoplastic neuroepithelial tumour,
Other = other pathology. Dash indicates no available pathology information.

Supplementary Table 1: **Metadata of EMU patients.** Patient identifiers are listed under "Subject." IEEG Portal patients (MC and HUP hospitals) have the same identifier as the one used by the database. Metadata was extracted from the reports provided on the IEEG Portal (MC and HUP patients) or the patient clinical reports (UCLH patients).

Subject	Age (yrs)	Sex	Age at diagnosis (yrs)	Lobe	Previous resection	# of seizures analysed	# of electrodes analysed	Total recording time (days)	Sampling frequency
NeuroVista 1	26	M	4	PT	No	94	15	767.4	400 Hz
NeuroVista 3	22	F	16	PT	Yes	273	16	557.5	400 Hz
NeuroVista 6	62	M	37	T	No	69	16	441.3	400 Hz
NeuroVista 7	52	M	26	FT	No	212	16	184.8	400 Hz
NeuroVista 8	48	M	20	FT	Yes	452	14	558.4	400 Hz
NeuroVista 9	51	F	10	OP	No	147	14	394.9	400 Hz
NeuroVista 10	50	F	15	FT	Yes	446	16	373.2	400 Hz
NeuroVista 11	53	F	15	FT	No	351	14	721.6	400 Hz
NeuroVista 13	50	M	20	T	Yes	425	16	746.9	400 Hz
NeuroVista 15	36	M	5	T	Yes	57	16	465.6	400 Hz

Sex
M = male, F = female

Lobe
T = temporal, F = frontal,
P = parietal, O = occipital,
IH = interhemispheric

Supplementary Table 2: **Metadata of NeuroVista patients.** Clinical metadata and patient demographics are reproduced from [2].

Subject	Total recording time	# seizures analysed	# electrodes analysed	Sampling frequency
**I004_A0001_D001 (Dog 1)**	45d 19h	86	16	399.6098 Hz
**I004_A0002_D001 (Dog 2)**	451d 20h	58	16	399.6098 Hz
**I004_A0003_D001 (Dog 3)**	475d 17h	43	15	399.6098 Hz

Supplementary Table 3: **Metadata of dogs.**

Supplementary Text 2 Distributions of seizure durations

The following figures show the seizure duration distribution within each subject as a histogram (Fig. Supplementary Figure 1). We show the distributions in duration, as well as natural log duration for reference. In general, we observed a wide range of seizures with different durations within most subjects.

Interestingly, we also observed that the variance of a duration distribution tended to grow with the mean of the distribution - a phenomenon also known as heteroscedasticity. We also show this in Fig. Supplementary Figure 2. Heteroscedasticity is generally not a desirable feature of the data for subsequent analysis, and we therefore log transformed the seizure duration. This transformation removed the heteroscedasticity in the data (Fig. Supplementary Figure 2). I.e., in a subject that has seizures of 40 seconds most of the time, a seizure of 20 seconds is unusual, whereas in a subject with seizures of mostly 3 minutes, a seizure of 2 minutes 40 seconds is more common. Therefore, instead of comparing absolute time differences (in this case 20 seconds for both), it is more informative to compare time ratios (i.e. difference of log time). Similarly, within

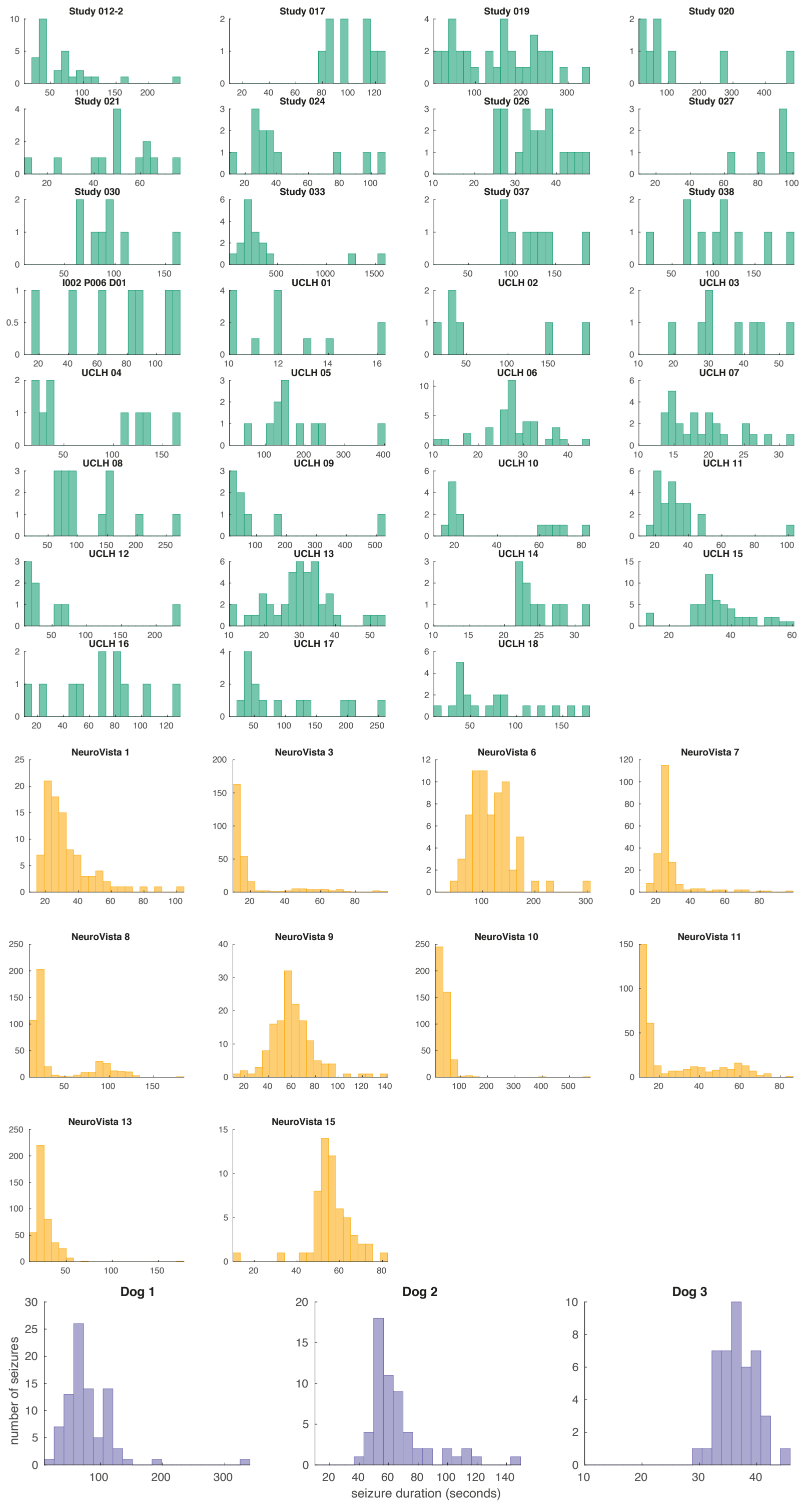

Supplementary Figure 1: **Seizure duration distribution in each subject.** x-axis shows duration in seconds, y axis shows the number of seizures.

subjects, the difference between 3 minutes and 2 minutes 40 seconds is also more expected than the difference between 40 seconds and 20 seconds.

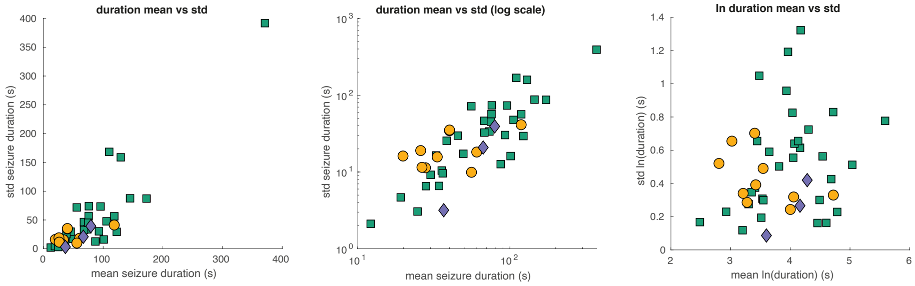

Supplementary Figure 2: **Heteroscedasticity in seizure duration data across subjects** Left: scatter plot of standard deviation (std) against the mean seizure duration in each subject. Middle: log scale plot of the same plot as left. Right: scatter plot of standard deviation (std) against the mean of the natural log of seizure duration in each subject. Marker convention as in Fig. 2J. Heteroscedasticity can be clearly seen in left and middle panels before log transformation of the duration. Heteroscedasticity is substantially reduced after transformation duration with natural log (right).

In Fig. Supplementary Figure 3, we also show the seizure duration distribution after log transformation. In Suppl. Supplementary Text 5, we additionally show that the range of seizure duration in a subject does not influence the relationship between seizure pathways and durations in that subject.

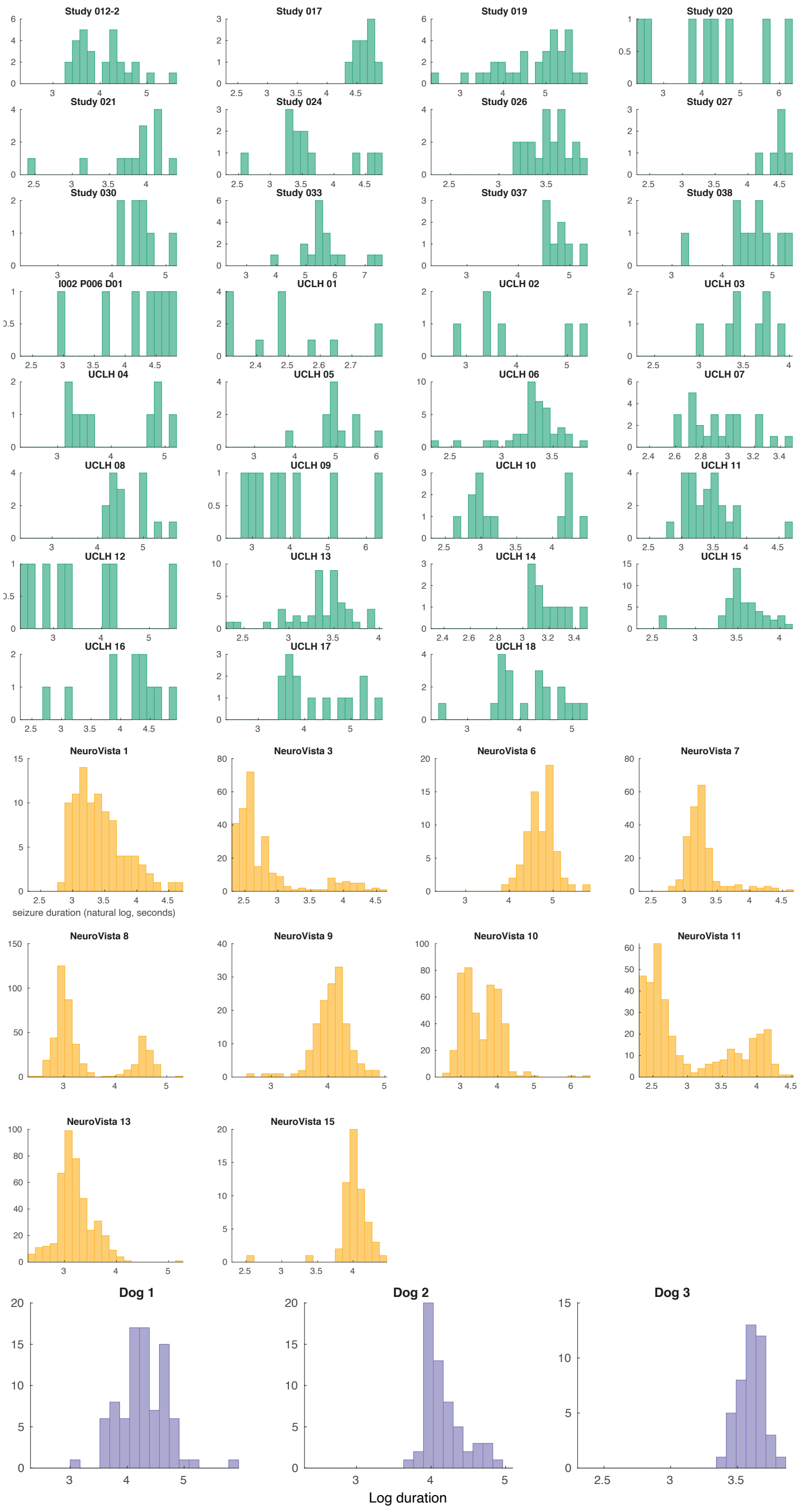

Supplementary Figure 3: **Seizure log duration distribution in each subject.** x-axis shows duration in natural log, y axis shows the count.

Supplementary Text 3 Dogs: identifying seizure terminations

For recordings from the dogs, seizure onset was determined using the seizure onsets provided on the IEEG Portal; however, seizure termination times were not marked in this dataset. Therefore,

seizure termination was identified algorithmically using an approach similar to [7]. For each dog, the time periods around each marked seizure onset were extracted, beginning with 300s before seizure onset and ending with sufficient time after seizure onset to capture all seizure terminations, based on visual inspection (460s for Dog 1, 250s for Dog 2, and 150s for Dog 3). Because identifying seizure termination relied on reference preictal data, dog seizures were only included in the analysis if 1) there was at least 300s between the seizure start and the termination of the previous seizure, and 2) if the preictal period, defined as three minutes to one minute before seizure start, lacked large noisy or missing segments.

After the preprocessing steps described in Methods section "Intracranial EEG preprocessing for epilepsy monitoring unit patients and dogs", we identified the time period containing seizure activity for each channel in each iEEG segment. Seizure activity was identified based on an increase in signal absolute slope, $S(t)$, compared to each seizure's preictal period. The absolute slope S of each channel i was given by

$$S_i(t) = |\tfrac{x_i(t)-x_i(t-1)}{\Delta t}|$$

where x_i is the time series voltage value of channel i and Δt is size of the time step between successive iEEG time points. $S_i(t)$ was then normalised to $S_i'(t)$ by dividing each time point by $\sigma_{i,pre}$, the standard deviation of the absolute slope of channel i during the seizure's preictal period, and smoothed by applying a 5s moving average sliding window. Channel i was considered epileptic at time point t if $S_i'(t)$ was greater than 2.5. Seizure termination was marked as the first time, following the clinically marked seizure start, when the number of epileptic channels fell below and remained below two channels for at least 1.5s.

Supplementary Text 4 Significance test results for pathway dissimilarities/duration differences correlations

Fig. Supplementary Figure 4 shows which correlations between pathway dissimilarites and duration differences were significant after FDR correction for multiple comparisons. The Mantel test was used to test the statistical significance of each correlation (see Methods, section "Correction for

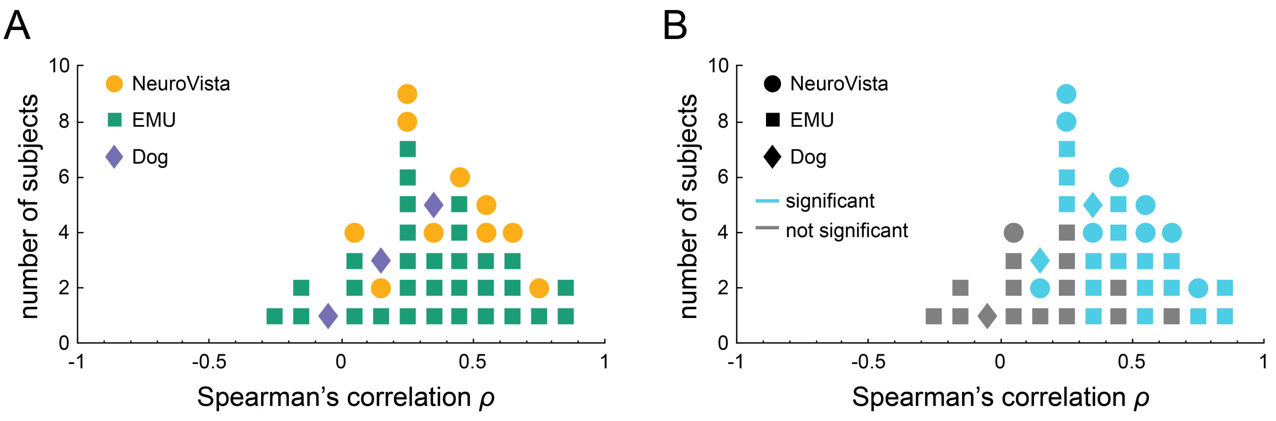

Supplementary Figure 4: **Significance test results for pathway dissimilarities/duration differences correlations.** A) Reproduction of Fig. 2J showing the distribution of correlations between pathway dissimilarities and duration differences across subjects. Each marker corresponds to a subject, with the marker shape and colour indicating the subject's cohort. B) The same distribution as in A, but with markers now coloured by whether the subjects correlation was significant after FDR correction for multiple comparisons.

multiple comparisons"). Note that the significance of each correlation depended not only on the strength of the correlation, but also the number of the seizures in each subject and the relationships between the subject's seizure pathways. As such, some relatively high correlations may not be significant and vice versa.

Supplementary Text 5 The relationship between pathway and duration variability does not depend on the range in either feature.

It is possible that the relationship between seizure pathways and durations requires the existence of a certain amount of variability in pathways and duration. For example, if seizure durations are highly consistent, then we would not expect there to be coinciding changes in seizure pathways. To test this hypothesis, we compared the correlation between pathway dissimilarities and duration differences to the maximum duration difference (Fig. Supplementary Figure 5A) and maximum pathway dissimilarity (Fig. Supplementary Figure 5B) in each subject. The maximum duration difference describes the greatest proportional change in seizure duration within each subject, while the maximum pathway dissimilarity captures the largest level of variability in the subject's seizure

pathways. In other words, these measures serves as ranges for each subject's seizure durations and seizure pathways.

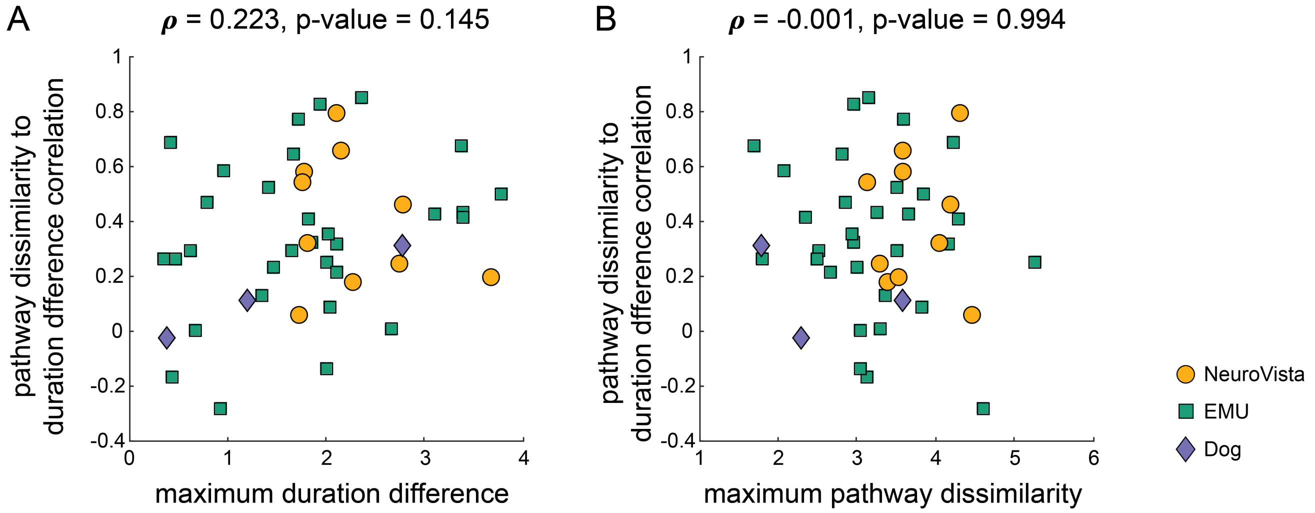

Supplementary Figure 5: **The relationship between pathway and duration variability does not depend on the range in either feature.** A) The correlation between pathway dissimilarities and duration differences plotted versus the maximum duration difference of each subject. The colour and shape of each marker corresponds to the subject's cohort. B) The correlation between pathway dissimilarities and duration differences plotted versus the maximum pathway dissimilarity of each subject.

In both cases, we found no significant relationship between the extent of variability and the correlation between pathway dissimilarities and duration differences. There was a weak, but insignificant, positive association between pathway dissimilarity/duration difference correlations and maximum durations differences. As such, there was a slight tendency for subjects with greater duration variability to have a stronger relationship between pathways and durations. Future work could investigate this relationship in a larger cohort. However, overall, the relationship between pathways and durations does not depend on the range of these features.

Supplementary Text 6 Prevalence and features of elasticity and semblance

Fig. Supplementary Figure 6 describes the prevalence, as well as the level of elasticity and semblance in each subject (see Methods, section "Defining elasticity and semblance", for definitions). Fig. Supplementary Figure 6A demonstrates that it was common for a high proportion of seizures

with similar pathways to have different durations (i.e., elasticity). Likewise, Fig. Supplementary Figure 6B reveals that in many subjects, seizures with similar durations had different pathways (i.e., semblance).

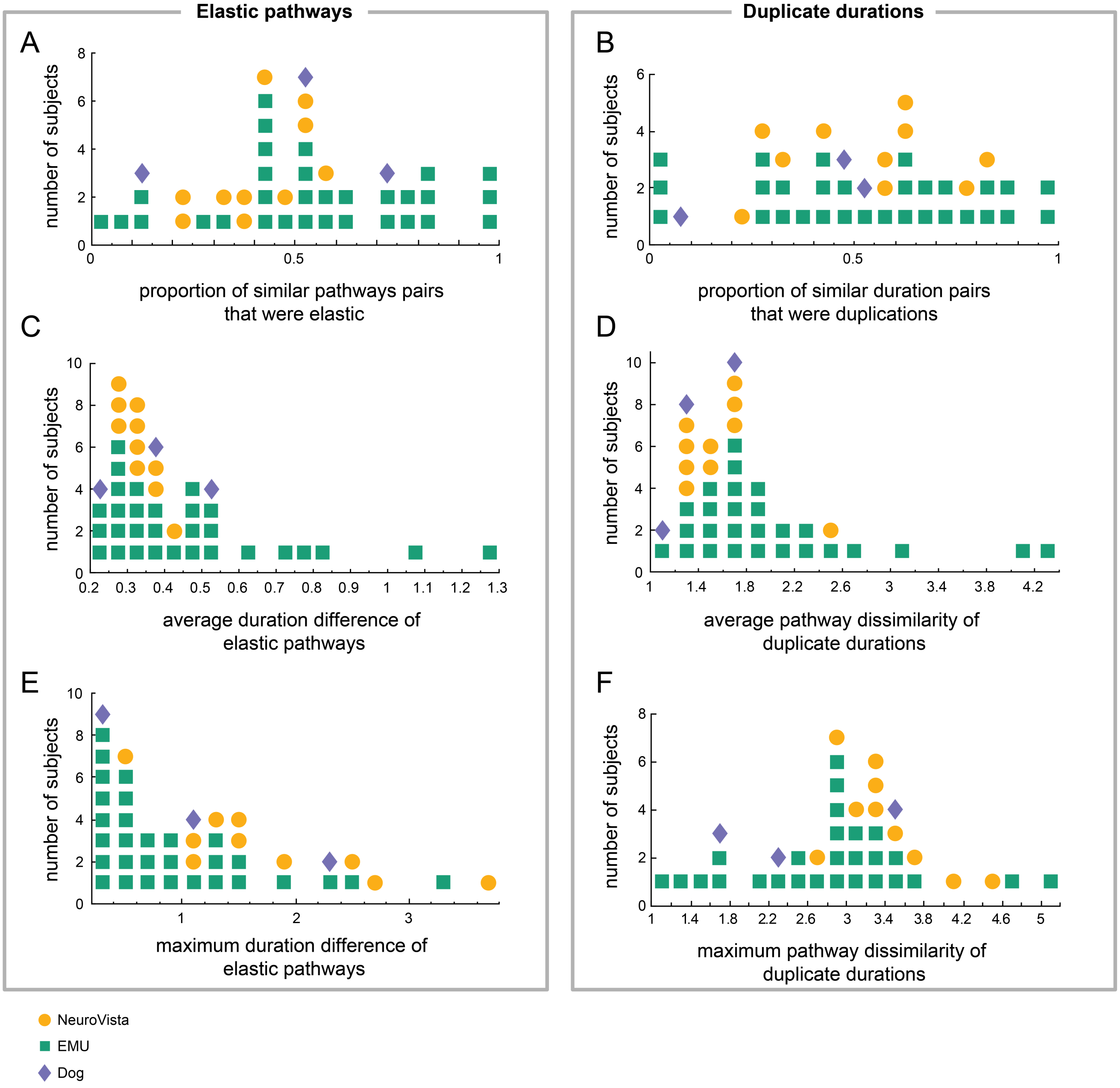

Supplementary Figure 6: **Prevalence and features of elasticity and semblance.** In all plots, markers correspond to subjects and their colour and shape indicates the subjects' cohorts. A) Proportion of seizure pairs with similar pathways that were elastic (i.e., that had different durations) in each subject. B) Proportion of seizure pairs with semblance (i.e., that had different pathways). C,E) In subjects with elasticity pairs, the median (C) and maximum (E) duration difference of the elastic pathways. D,F) In subjects with semblance pairs, the median (D) and maximum (F) pathway dissimilarity of the semblance.

The level of pathway elasticity, which can be quantified by the duration differences of the elastic seizure pairs, varied across subjects (Fig. Supplementary Figure 6C,E). On average, duration differences of 0.2 to 0.55 (equivalent to a $e^{0.2}$ to $e^{0.55} = 1.22$ to 1.73 fold change in seizure duration), were common, but extremes of $e^1 = 2.72$ or higher were also observed in many subjects. Thus, seizures with similar pathways could have drastically different durations. Likewise, the level of pathway dissimilarity between seizure pairs with similar durations varied across subjects (Fig. Supplementary Figure 6D,F). Average pathway dissimilarities of approximately 1.2 to 2 were common, but higher dissimilarities of 3 or higher were also observed in many subjects. Therefore, seizures with similar durations could have very different pathways.

Supplementary Text 7 Comparison of duration populations and pathway dissimilarities

Fig. Supplementary Figure 7 shows the adjusted Rand indices (Fig. Supplementary Figure 7A,B) and Rand indices (Fig. Supplementary Figure 7C,D) between duration populations and pathway clusters in subjects with two duration populations (see Methods). The significance of each index for will depend on the index value as well as the number of seizures and cluster sizes. The Rand index is the proportion of seizure pairs that have the same relationship in both partitions (i.e., in the same cluster in both partitions or in different clusters in both partitions), and is therefore easily interpretable. However, the Rand Index greatly depends on the relative cluster sizes, making the adjusted Rand index a better measure for understanding the strength of the agreement of the two partitions. We therefore provide both measures here to evaluate the agreement between duration populations and pathway clusters.

Supplementary Text 8 Clinical metadata comparisons

To determine if patterns of pathway and duration variability were related, we compared nine subject-specific pathway and duration measures to four clinical variables in the EMU patients. The pathway and duration measures were

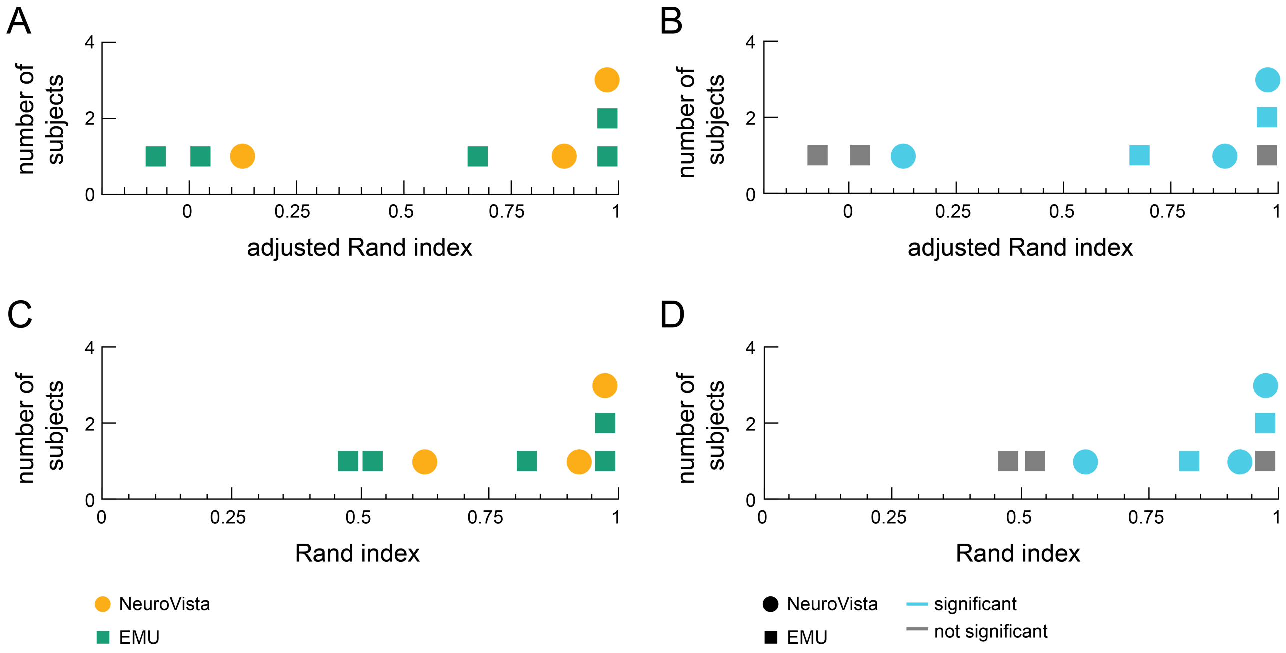

Supplementary Figure 7: **Comparison of duration populations and pathway dissimilarities.** A) Reproduction of Fig. 6 I showing the distribution of adjusted Rand indices between duration populations and pathway clusters. Marker colour and shape correspond to each subject's cohort. B) Same as A, but marker colour indicates whether the adjusted Rand index of the subject was significant after correction for multiple comparisons. C-D) The corresponding Rand indices of the same subjects.

1. The correlation between the subject's pathway dissimilarities and duration differences.
2. The subject's maximum duration difference.
3. The subject's median duration difference.
4. The subject's longest seizure.
5. The subject's median seizure duration.
6. The proportion of the subject's elasticity seizure pairs.
7. The proportion of the subject's semblance seizure pairs.
8. The median duration difference of the subject's elasticity seizure pairs.
9. The median pathway dissimilarity of the subject's semblance seizure pairs.

These measures were compared to

1. The patients' International League Against Epilepsy (ILAE) surgical outcomes ($n = 26$).
2. The patients' disease durations ($n = 31$).
3. Whether the patient had temporal ($n = 12$) or extratemporal ($n = 15$) epilepsy.
4. Whether the patient had seizures with left ($n = 15$) or right ($n = 13$) hemisphere onset.

ILAE surgical outcomes and disease duration were associated with the variability measures using Spearman's correlation. The variability measures of temporal versus extratemporal patients and left versus right hemisphere onset patients were compared using Wilcoxon rank sum tests.

There were no significant relationships between seizure pathway/duration measures and surgical outcome after FDR correction for multiple comparisons, although there was a trend (Spearman's correlation $\rho = 0.45, p = 0.0209$) for surgical outcome to worsen as median seizure duration increased. This association may have been driven by other clinical factors that influence both surgical outcome and seizure duration, such as whether a patient has focal to bilateral tonic-clonic seizures [1, 4, 5] and the localisation of the epileptogenic zone [3, 6]. There were also no significant differences between patients with different onset locations, and the only trend was for patients with temporal onset to have higher median seizure duration than patients with extratemporal onset (Wilcoxon rank sum test, $p = 0.0381$), consistent with previous findings [6].

Disease duration was significantly inversely correlated to median seizure duration (Spearman's correlation $\rho = -0.56, p = 0.0011$), with median seizure duration decreasing as disease duration increased. Additionally, the tendency of similar seizure pathways to be elastic significantly decreased with disease duration (Spearman's correlation $\rho = -0.51, p = 0.0038$). Disease duration at the time of the iEEG recording was not sampled at a random time for each patient, but determined by the clinical decision to undergo presurgical monitoring. Therefore, disease duration in our cohort could also be associated with clinical considerations such as seizure severity and the patient's level of antiepileptic medication resistance. As such factors could also influence measures such as seizure duration, it is difficult to interpret the observed associations with disease duration.

Supplementary Text 9 Contribution of different frequency bands to seizure pathways

In this supplementary analysis, we investigated the relative contribution of each frequency band to dissimilarities in seizure pathways. We turned to our NMF components (Fig. Supplementary Figure 8), which provided the basic building blocks of all seizures in a subject in terms of their seizure functional connectivity. Each seizure in a given subject is composed of a sequence of time windows, where in each time window, the functional connectivity can be reconstructed as a (sparse) linear combination of these components. Effectively, the seizures can be understood as a pathway through the space spanned by these components. This representation has the advantage of capturing the sequence of functional connectivity over the duration of a seizure with minimal loss of information. It is also a general representation in terms of pairwise interactions between channels in each frequency band (e.g. it can faithfully represent seizures that slowly spread to different regions, as well as seizures that never spread from onset, or diffuse onset seizures, etc.). It is, however, important to note this representation is not directly clinically interpretable, and may highlight pairwise interactions that are not salient in the visual impression of the EEG.

In an example subject (Fig. Supplementary Figure 8), we can see that different frequency bands contribute to different components, indicating that several frequency bands were relevant to this subject's seizures. Moreover, multiple frequency bands contribute to changes across frequency bands. To quantify the contribution of different frequency bands to pathway dissimilarities, we calculated the pairwise difference between components as cityblock distance and normalised this difference to 1 across frequency bands, yielding a relative contribution of each frequency band to the component distances. (Fig. Supplementary Figure 9). If components represented changes in seizure functional connectivity in a single frequency band, we would see the largest pairwise differences between components in a single frequency band, which is not the case for this subject. This example patient is qualitatively representative of all subjects, and the median relative contributions of each frequency band were approximately equal in most subjects. Even the outlier maximum relative contribution of a single frequency band did not exceed 50% (Fig. Supplementary Figure 10).

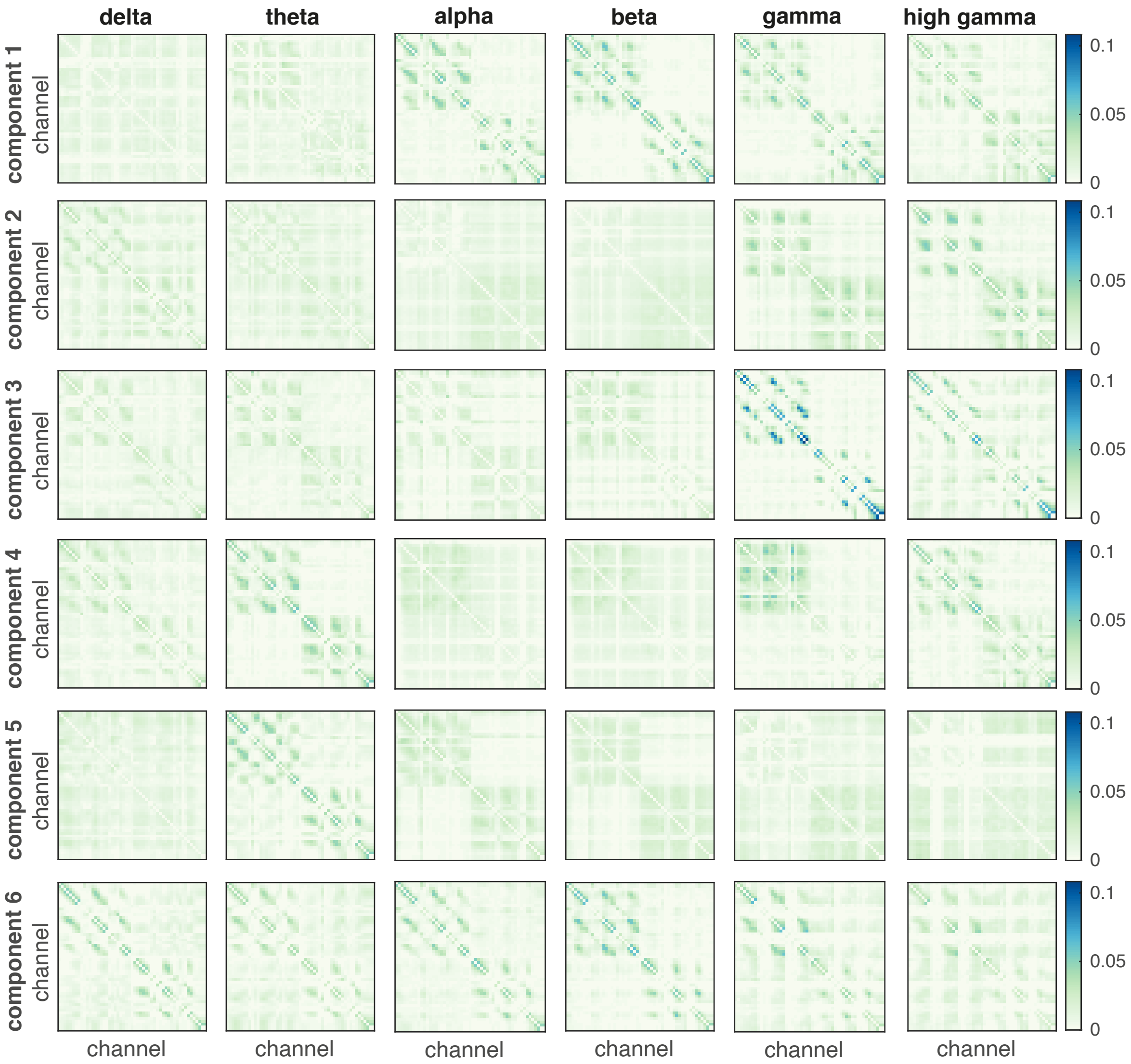

Supplementary Figure 8: **Non-negative matrix factorisation components for example EMU subject UCLH 10.** Each component is comprised of functional connectivity across six frequency bands. Each seizure functional connectivity at each time window can be reconstructed as a (sparse) linear combination of these components.

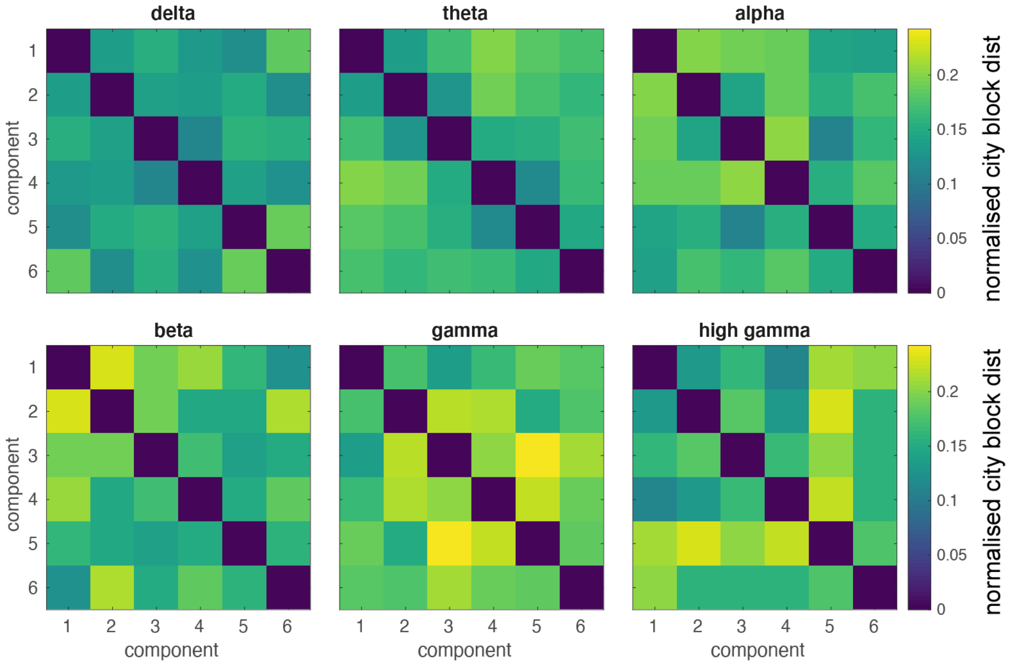

Supplementary Figure 9: **Pairwise differences of components example EMU subject UCLH 10.** Pairwise cityblock distance is show for example EMU subject UCLH 10 normalised across frequency bands to yield a measure of relative contribution of each frequency band to this subject's seizure pathways.

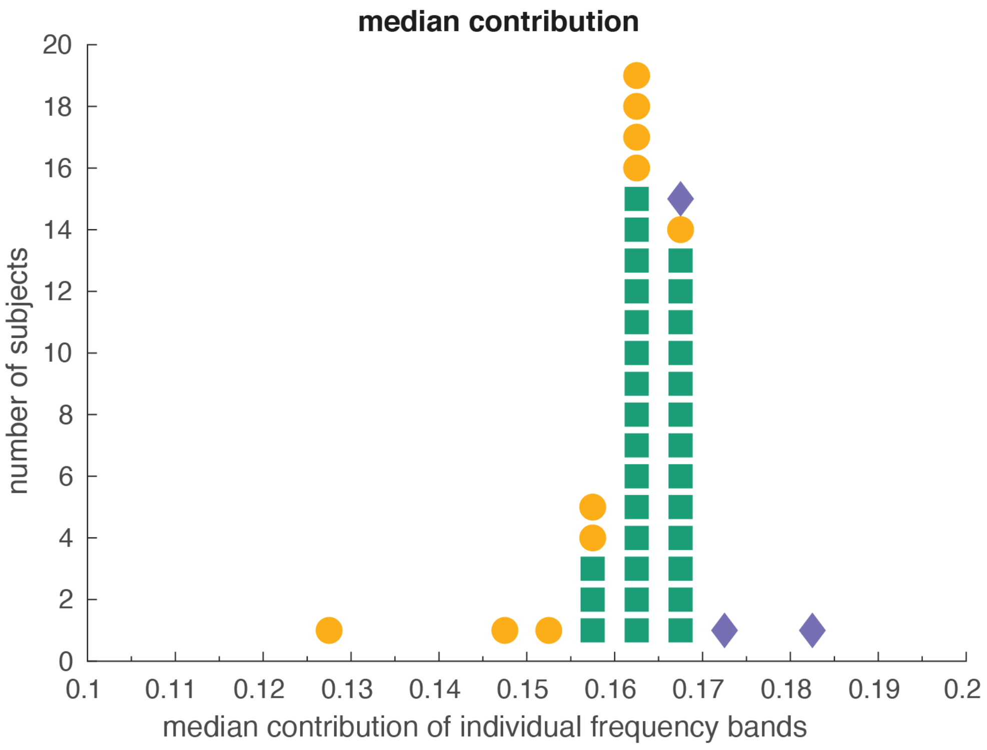

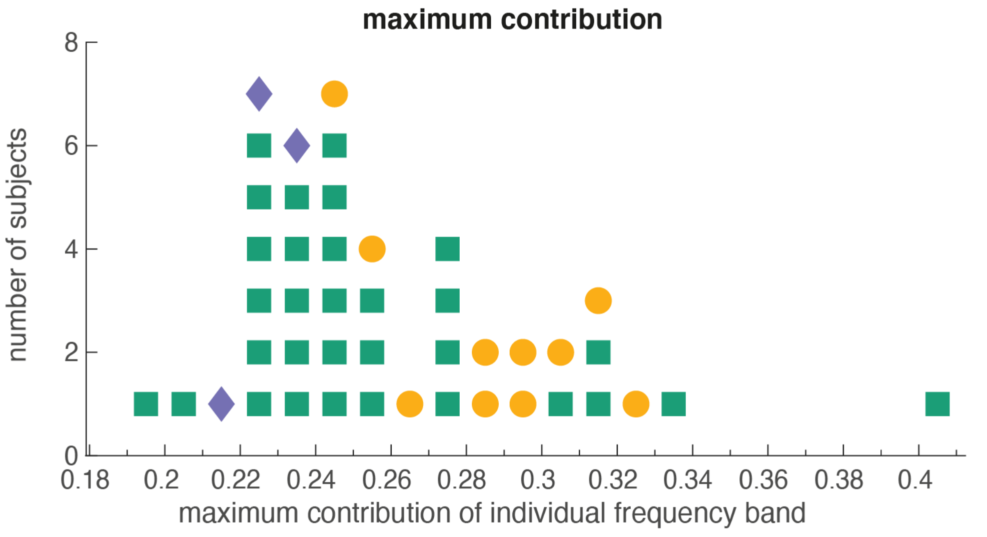

Supplementary Figure 10: **Median and max relative contribution of frequency bands in all subjects.** Dot plot of median and max relative contribution of frequency bands. Most medians are tightly centred around 0.16 ($= \frac{1}{6}$) indicating equal contribution of all bands.

References

[1] Maxime O. Baud, Serge Vulliemoz, and Margitta Seeck. Recurrent secondary generalization in frontal lobe epilepsy: predictors and a potential link to surgical outcome? *Epilepsia*, 56(9):1454–1462, 2015. ISSN 00139580. doi: 10.1111/epi.13086. URL `http://doi.wiley.com/10.1111/epi.13086`.

[2] Mark J. Cook, Terence J. O'Brien, Samuel F. Berkovic, Michael Murphy, Andrew Morokoff, Gavin Fabinyi, Wendyl D'Souza, Raju Yerra, John Archer, Lucas Litewka, Sean Hosking, Paul Lightfoot, Vanessa Ruedebusch, W. Douglas Sheffield, David Snyder, Kent Leyde, and David Himes. Prediction of seizure likelihood with a long-term, implanted seizure advisory system in patients with drug-resistant epilepsy: A first-in-man study. *The Lancet Neurology*, 12:563–571, 2013. ISSN 14744422. doi: 10.1016/S1474-4422(13)70075-9.

[3] Jane de Tisi, Gail S. Bell, Janet L. Peacock, Andrew W. McEvoy, William Fj Harkness, Josemir W. Sander, and John S. Duncan. The long-term outcome of adult epilepsy surgery, patterns of seizure remission, and relapse: a cohort study. *The Lancet*, 378:1388–1395, 2011. ISSN 01406736. doi: 10.1016/S0140-6736(11)60890-8. URL `http://dx.doi.org/10.1016/S0140-6736(11)60890-8`.

[4] Judith Dobesberger, Aleksandar J. Ristić, Gerald Walser, Giorgi Kuchukhidze, Iris Unterberger, Julia Höfler, Edda Amann, and Eugen Trinka. Duration of focal complex, secondarily generalized tonic-clonic, and primarily generalized tonic-clonic seizures - A video-EEG analysis. *Epilepsy and Behavior*, 49:111–117, 2015. ISSN 15255069. doi: 10.1016/j.yebeh.2015.03.023.

[5] Elisabeth Kaufmann, Magdalena Seethaler, Michael Lauseker, Min Fan, Christian Vollmar, Soheyl Noachtar, and Jan Rémi. Who seizes longest? Impact of clinical and demographic factors. *Epilepsia*, 61:1376–1385, 2020. ISSN 15281167. doi: 10.1111/epi.16577.

[6] Daeyoung Kim, Jae-Wook Cho, Jihyun Lee, Eun Yeon Joo, Seung Chyul Hong, Seung Bong Hong, and Dae-Won Seo. Seizure Duration Determined by Subdural Electrode Recordings

in Adult Patients with Intractable Focal Epilepsy. *Journal of Epilepsy Research*, 1(2):57–64, 2011. ISSN 2233-6249. doi: 10.14581/jer.11011.

[7] Kaspar Schindler, Howan Leung, Christian E. Elger, and Klaus Lehnertz. Assessing seizure dynamics by analysing the correlation structure of multichannel intracranial EEG. *Brain*, 130: 65–77, 2007. ISSN 00068950. doi: 10.1093/brain/awl304.
